# A micro-XRT image analysis and machine learning methodology for the characterisation of multi-particulate capsule formulations

**DOI:** 10.1016/j.ijpx.2020.100041

**Published:** 2020-01-15

**Authors:** Frederik J.S. Doerr, Alastair J. Florence

**Affiliations:** a EPSRC CMAC Future Manufacturing Research Hub, Technology and Innovation Centre, 99 George Street, Glasgow G1 1RD, UK; b Strathclyde Institute of Pharmacy & Biomedical Sciences (SIPBS), University of Strathclyde, Glasgow G4 0RE, UK

**Keywords:** Pharmaceutical formulation, Micro-XRT particle analysis, Watershed image segmentation, Sensitivity analysis, Feature selection, Machine learning, Classification model, Abbreviation, Description, IEV, Translation- and rotation-invariant cross-section, OC-SVM, One-class support vector machine, OSH, Optimal separating hyperplane, RBF, Radial basis function, ROI, Region-of-interest, TC-SVM, Two-class support vector machine, V, Single pellet, V_ROI, Single pellet region-of-interest, V_CS, Capsule shell, V_CS_ROI, Capsule shell region-of-interest, V_CS_InV, Capsule shell internal volume, V_CS_Poros, Capsule shell void, V_CP, Pellet population, V_CP_ROI, Pellet population region-of-interest, V_CP_Poros, Pellet population porosity

## Abstract

The application of X-ray microtomography for quantitative structural analysis of pharmaceutical multi-particulate systems was demonstrated for commercial capsules, each containing approximately 300 formulated ibuprofen pellets. The implementation of a marker-supported watershed transformation enabled the reliable segmentation of the pellet population for the 3D analysis of individual pellets. Isolated translation- and rotation-invariant object cross-sections expanded the applicability to additional 2D image analysis techniques. The full structural characterisation gave access to over 200 features quantifying aspects of the pellets' size, shape, porosity, surface and orientation. The extracted features were assessed using a ReliefF feature selection method and a supervised Support Vector Machine learning algorithm to build a model for the detection of *broken* pellets within each capsule. Data of three features from distinct structure-related categories were used to build classification models with an accuracy of more than 99.55% and a minimum precision of 86.20% validated with a test dataset of 886 pellets. This approach to extract quantitative information on particle quality attributes combined with advanced data analysis strategies has clear potential to directly inform manufacturing processes, accelerating development and optimisation.

## Highlights

• Coupling micro-XRT analysis with feature selection and machine learning for advanced pharmaceutical product characterisation.
• Information on particle 3D-orientation were utilised to extract translation- and rotation-invariant object cross-sections.
• Successful extraction of over 200 quantitative pellet descriptors linked to size, shape, porosity, surface and orientation.
• Sensitivity analysis and ReliefF feature selection approach to identify predictive features for pellet classification.
• Feature-based binary SVM classification model for the detection of broken pellets within the formulated system.

## Graphical abstract

**Figure:**
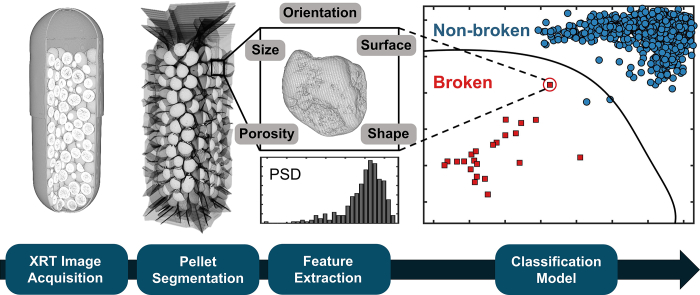

## 1 Introduction

The measurement of morphological descriptors and structural properties is widely applied in the pharmaceutical industry to evaluate quality attributes of solid products that affect their performance in the manufacturing process or upon final administration to the patient. Methods to aid understanding the effect of process and material parameters on the final product characteristics enable a Quality by Design (QbD) approach to achieve consistent, safe and effective quality products (Yu, 2008). The diversity of structure-related critical quality attributes has led to a broad range of analytical techniques being used to investigate size, shape and porosity of pharmaceutical products and drug product intermediates (Fonteyne et al., 2014; Markl et al., 2018). Employed analysis techniques are often limited to bulk information or to the characterisation of individual particles when working with multi-particulate systems. Commonly, multiple complementary techniques are required to achieve a full characterisation of all the relevant structural properties (Michie et al., 2012; Brown et al., 2018). Often the techniques are destructive or require larger quantities of sample material for a successful characterisation.

X-ray microtomography (micro-XRT) can be applied to investigate non-destructively a wide range of particle properties for (multi-) particle systems. The reconstructed cross-sections of the micro-XRT image data allow three dimensional visual inspections of the scanned object. The smallest structural attribute that can be resolved typically requires multiple pixels/voxels (volume pixel) due to the partial volume effect which describes the blurring of intensity edges in digital micro-XRT images (Soret et al., 2007). Nowadays, commercial micro-XRT systems commonly achieve spatial resolutions of around 1 μm - 10 μm which corresponds to the projected sample size on a minimum of 3–5 pixels (Wildenschild and Sheppard, 2013; Schoeman et al., 2016). Image processing capabilities can be further used to extract quantitative information simultaneously on bulk and single particle properties. Specific applications of micro-XRT with subsequent image processing have been reported for (multi-) particle systems targeting structural features related to particle size (Doerr et al., 2018; Fang et al., 2017), surface (Doerr et al., 2018; Fang et al., 2017), morphology (Doerr et al., 2018; Fang et al., 2017; Zheng and Hryciw, 2015; Zhou et al., 2017; Su and Yan, 2018; Asachi et al., 2018), porosity (Doerr et al., 2018; Fang et al., 2017; Dadkhah and Tsotsas, 2014; Farshchi et al., 2019) and attributes of the local micro-structure (Perfetti et al., 2010; Sondej et al., 2015). The image processing strategies are often tailored to meet specific analysis requirements to further relate individual sample properties to the manufacturing process or product performance (Asachi et al., 2018; Perfetti et al., 2010). The quality of the collected micro-XRT image data and the applied image processing algorithms can both have an impact on extracted quantitative information (Yip and Aerts, 2016). A sensitivity analysis allows the user to evaluate the risk for data variability with changing parameters during collection and image processing which has been described in details elsewhere (Al-Sarraf et al., 2008).

Extracted quantitative information from collected micro-XRT image data can be exploited to inform machine learning (ML) models which are designed to provide answers to complex questions involving automated decision making processes without the need to provide explicit instructions. ML approaches can be used effectively to identify patterns and inference within large feature-based datasets (Hastie et al., 2009). Support Vector Machines (SVMs) are a class of supervised ML algorithms originally designed to solve binary classification problems to define decision boundaries in multidimensional feature datasets (Hastie et al., 2009; Boser et al., 1992). SVM models have since been utilised for a wide range of applications including solubility prediction (Cheng et al., 2011) or crystal shape classification (Ochsenbein et al., 2015), analysis of biomedical data (Dreiseitl et al., 2010; Mourão-miranda et al., 2011) and network anomaly detection in computer science (Perdisci et al., 2006).

For the first time, this study employs a micro-XRT system to access over 200 structural features for a pharmaceutical multi-particulate product and presents strategies for the extraction and evaluation of 3D quantitative morphological descriptors by means of image processing and analysis. The introduction of translation- and rotation-invariant object cross-sections additionally enables the reliable application of 2D image analysis methodologies. The extracted features are used to solve a classification problem for the detection of *broken* pellets within the pellet population of each sample. The selection of reliable structural features for this classification problem included a micro-XRT sensitivity analysis to assess the impact of image quality and image processing parameters as well as a feature selection approach using the popular ReliefF method described in detail elsewhere (Kira and Rendell, n.d.; Kononenko et al., 1997; Robnik-Šikonja and Kononenko, 1997; Robnik-Šikonja and Kononenko, 2003). The micro-XRT investigation of particle systems is demonstrated on commercially available capsules containing formulated ibuprofen pellets for oral administration.

## 2 Materials and methods

### 2.1 Commercial ibuprofen capsule

Capsules with ibuprofen pellets for sustained drug-release (Galpharm Healthcare Ltd., Lot# 020288) were used as a model pharmaceutical multi-particulate system. The pellets of each capsule consist of 200 mg ibuprofen formulated with micro-crystalline cellulose, Eudragit NE30D, hypromellose, talc and colloidal silicon dioxide. The capsule shell is made from gelatin, titanium dioxide (E171), patent blue V (E131) and erythrosine (E127). In total, six capsules were investigated during this study. Three capsules were used for model training (references DTR: C0, C1 and C2) with three additional capsules used to generate the test dataset (references DTT: C3, C4 and C5).

### 2.2 X-ray tomography

A Skyscanner 2211 X-ray tomograph (NanoCT, Bruker, Kontich, Belgium) with cone-beam arrangement was employed for micro-XRT data collection. The samples were scanned with an image pixel size of 2.5 μm - 6 μm, frame averaging of 3 and a rotation step size of 0.2° (details listed in Table S1, ESI, page S1). The acquisition settings were varied in order to assess the impact of data acquisition parameters on the pellet classification model described in Section 2.4. The capsules C0 and C3 were scanned with high image quality. For all other capsules the data collection was optimised to reduce overall data acquisition times whilst accepting a decreased sample resolution (C1, C2, C4, C5). The X-ray acceleration voltage was 40 keV. A reference scan was collected at the end of each run to enable post-alignment correction and therefore compensate for potential shifts during the scan. Image reconstruction included beam hardening corrections and ring artefact reduction which were performed using NRecon with InstaRecon (version 1.7.1.6, Bruker, Kontich, Belgium). Visualisations of the image stacks were rendered using CTVox (version 3.2.0, Bruker, Kontich, Belgium).

### 2.3 3D image processing and single particle analysis

Micro-XRT image processing and analysis strategies were implemented in MATLAB R2018a (version 9.4.0.813654, MathWorks, United States). Structural features were extracted globally for the full sample from each capsule and for each individual pellet within the capsules.

Global features were defined as the capsules dimensions (i.e. max. Length, mean diameter) and internal porosity distribution. The measured porosities were further separated in order to distinguish inter- and intra-pellet porosity. This required a custom-made script to establish a region-of-interest (ROI) for the pellet distribution (V_CP_ROI) using a 3D-morphological closing operation and a secondary enclosed background filter integrated in a feedback-loop for detected internal background volumes previously described to define single particle ROIs (Doerr et al., 2018). Voxel-based arithmetic operations were used to calculate the internal capsule volume (V_CS_InV), the total intra-pellet porosity (V_CP_Poros) and inter-pellet void space (V_CS_Poros) of the capsule sample. Details on the calculations are included in Section S1.2 (ESI, page S2).

**Fig. 1:**
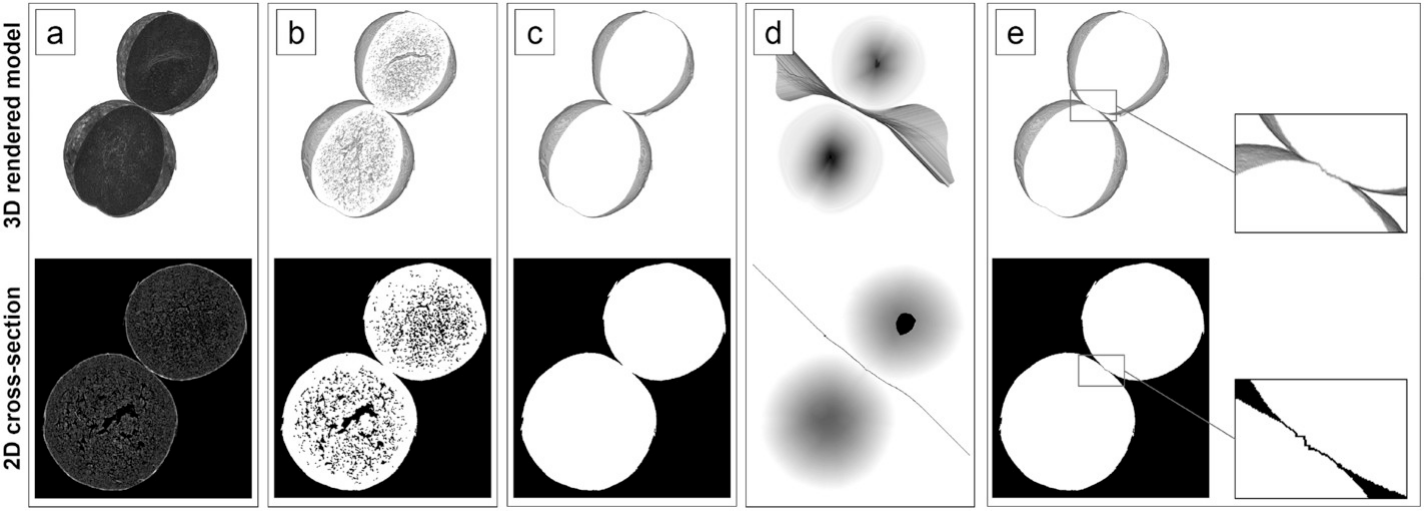
Image processing sequence to separate connected particle volumes. (a) Raw image data, (b) image binarization, (c) particle ROI, (d) marker-controlled watershed segmentation using a distance transformation with superimposed region markers and (e) separated particles.

A marker-controlled watershed transformation algorithm (Meyer, 1994) was applied to separate all connected “touching” primary objects within V_CP_ROI and to allow a subsequent structural analysis of each individual pellet. The image processing workflow for a successful volume segmentation using a marker-controlled watershed transformation of two connected objects is shown in Fig. 1. Reconstructed image cross-sections (Fig. 1a) are initially pre-processed using an edge-preserving local contrast image filter to reduce random image noise on grayscale. Image binarization (Fig. 1b) is performed with a histogram-based thresholding algorithm to convert the images to a monochromatic logic-mask (Ridler and Calvard, 1978). Remaining noise in the created binary image stack is removed with a series of noise reduction algorithms including a 3D sweep operation for non-connected binary object volumes. The image ROI (V_CP_ROI, Fig. 1c) is transformed into an Euclidean distance map with primary object markers superimposed in the local 3D minima (Fig. 1d). The distance-based grayscale image stack allows the successful application of the marker-supported watershed transformation to achieve a separation of the primary objects (Fig. 1e).

**Fig. 2:**
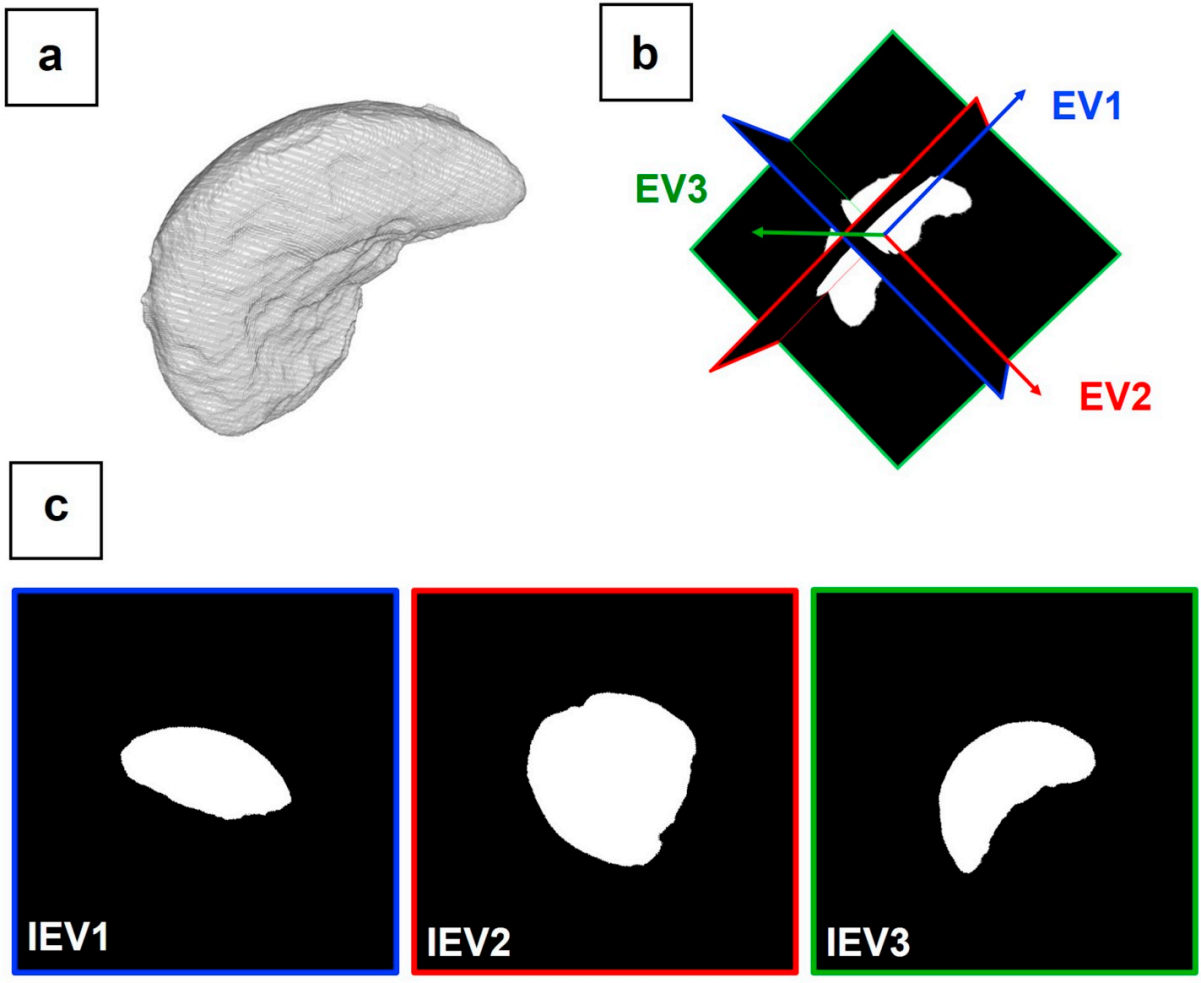
3D-to-2D image transformation: (a) The image eigenvectors are calculated from the 3D particle model (V_ROI) based on its second central image moment. (b) The 3D stack is re-sliced along the planes normal to the eigenvectors. (c) Three characteristic translation- and rotation-invariant 2D cross-sections (IEV1 - IEV3) are isolated [coloured high-resolution figure available online].

The segmented pellets from each capsule were further processed and analysed individually to extract a total of 206 features related to size, shape, porosity, surface and orientation. A detailed overview of the extracted features is provided in Table S2 (ESI, page S2). Basic features are derived from the evaluation of image moments (Hu, 1962). The zeroth order image moment for a binary digital image gives the total object voxel volume (3D). The first order moments contain information on the object centroid. The central moments include the components of the image centroid to provide translation invariance. The second order central moments allow the extraction of information about the object orientation within the 3D image space. Information on the object orientation and location were further used to isolate translation- and rotation-invariant 2D cross-sections (IEV) normal to the eigenvectors of an ellipsoid with matching second order central moment. For this 3D-to-2D transformation, the eigenvectors were utilised to consistently re-slice the 3D image stack as visualised in Fig. 2 independent of the pellet position and its 3D orientation. These 2D cross-sections (IEV1 - IEV3) enabled additional applications for feature extraction with established 2D image algorithms. 162 features of the total 206 pellet features are extracted from the analysis of the images IEV1 - IEV3.

### 2.4 Feature-based particle classification: relieff and SVM

A feature-based particle classification model was implemented to detect *broken* objects within the population of pellets. The ReliefF algorithm is a filter-method approach which can be used in conjunction with a labelled training dataset for the selection of features in categorical multidimensional classification problems (Kira and Rendell, n.d.; Kononenko et al., 1997; Robnik-Šikonja and Kononenko, 1997; Robnik-Šikonja and Kononenko, 2003). ReliefF ranks features according to their correlation with a labelled training dataset using a k nearest neighbors method. Extracted features were evaluated to identify those with the highest predictive power for this binary object classification problem: *broken* versus *non-broken* pellets. The ReliefF feature selection method was available in MATLAB and was performed using the training dataset (DTR). The number of nearest neighbors was selected to include the maximum number of labelled training objects providing class balance (k = min([# *non-broken*, # *broken*]) = 23). Including all observations of the minority class ensures maximum robustness against noise, but limits the detection of feature dependencies in the context of nearest neighbor locality to the majority class (Kononenko et al., 1997).

SVMs were employed to build a feature-based object classification model in order to identify *broken* pellets within the population (Hastie et al., 2009). The n-dimensional training dataset (DTR, each observation *x*_*i*_ ∈ *R*^*n*^) was used to build One-Class and Two-Class SVM models in MATLAB. The feature data were standardized to avoid scale effects on the classification outcome. The employed SVM kernel function was a radial basis function (RBF) to better adapt the optimal separating hyperplane (OSH) to non-linear data distributions. The One-Class SVM (OC-SVM) model was generated only considering observations of the *non-broken* pellets in the training dataset to create a close decision boundary around the majority class. The Two-Class SVM (TC-SVM) model was built using soft margins and prior probabilities proportional to the class membership distribution in DTR (*broken*:*non-broken*,1:38). The kernel scale and the box constraints for the TC-SVM model were selected automatically employing a Bayesian strategy for global optimisation using 4-fold cross-validation (Hastie et al., 2009).

## 3 Results and discussion

**Fig. 3:**
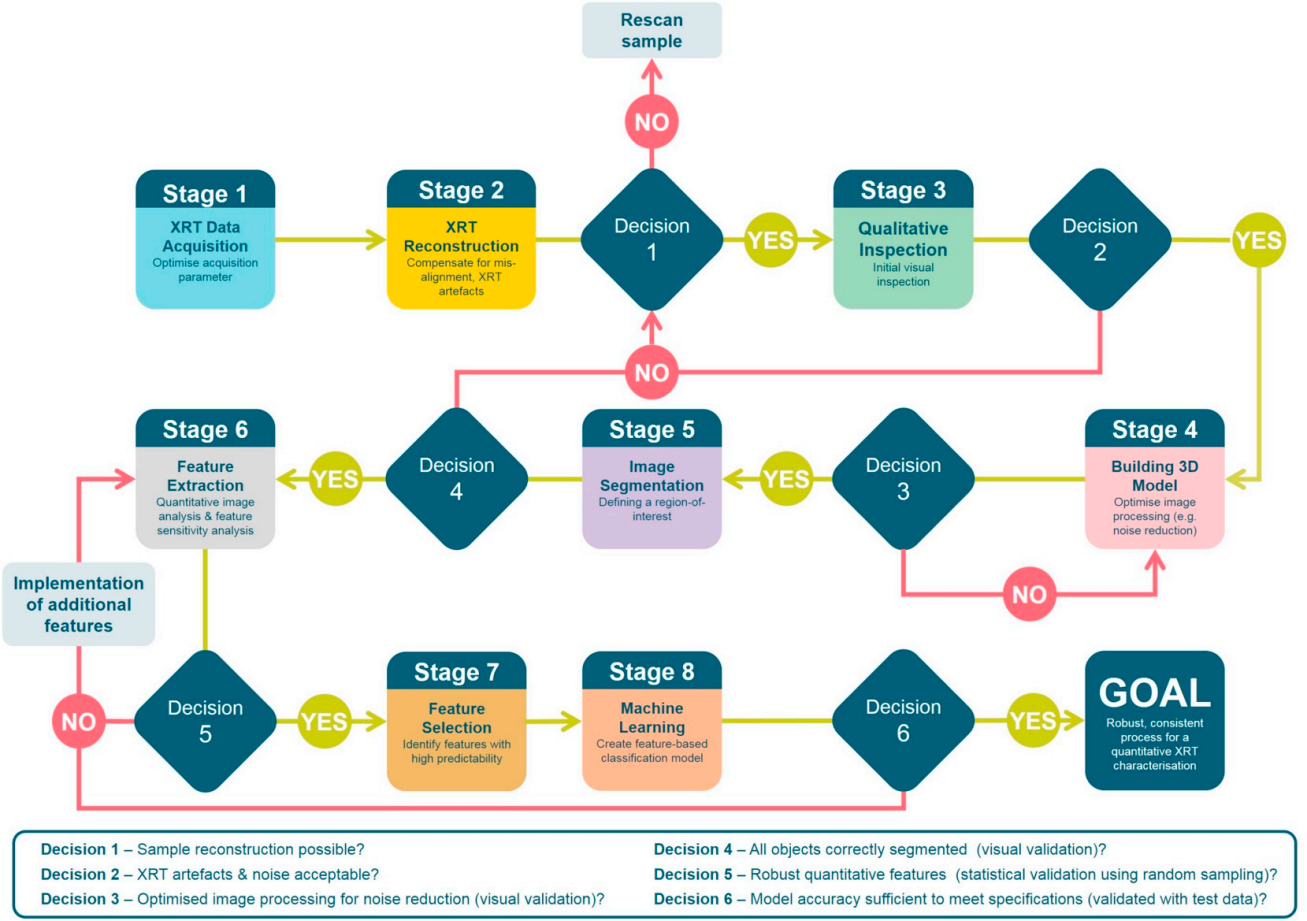
Full workflow combining micro-XRT analysis and machine learning methodologies for the characterisation of multi-particulate capsule formulations [coloured high-resolution figure available online].

The micro-XRT characterisation of each sample consists of basic steps of micro-XRT data acquisition, micro-XRT image reconstruction and an initial optimisation of image processing parameters for noise reduction (Fig. 3, *Stage 1–4*). This publication focuses on the subsequent analysis of the micro-XRT image data. Specific to this sample, the ibuprofen capsules were initially investigated to describe the multi-particulate system with specific measurements of size and overall porosity distribution (see Section 3.1). The segmentation of the pellet population permits an in-depth assessment of the structural attributes for each isolated pellet related to properties describing object size, shape, porosity, surface and orientation (Fig. 3, *Stage 5–6*, see Section 3.2 - Section 3.3). All extracted features were assessed as part of a sensitivity analysis to evaluate the feature robustness against changes in micro-XRT image quality and image processing parameters (Fig. 3, *Stage 6*, see Section 3.3). Three features were further identified with a ReliefF feature selection approach and used within a SVM classification model to detect *broken* pellets (Fig. 3, *Stage 7–8*, see Section 3.4). The features were selected to target distinct attributes of the pellet related to its size, shape and surface.

### 3.1 Capsule - full sample analysis

The dataset C0 was initially investigated using two manually defined regions-of-interest of the capsule shell (V_CS_ROI) and the pellet population (V_CP_ROI). This initial analysis exemplifies potential applications of micro-XRT extracting information on a global sample level to obtain an overview of key characteristics for this multi-particulate system. The results are listed in Table 1. They include a quantification of the sample's volume distribution as well as measurements of selected sample attributes which are linked to the capsule production process. Whilst beyond the scope of this study, this information could ultimately be used for process optimisation and/or to support a predictive framework, for instance to assess the product's final performance (Wilson and Crowley, 2011).

**Table 1:** Quantification of capsule volume distribution and individual measurements including capsule ROI surface area (A_S, V_CS_ROI_), pellets ROI surface area (A_S, V_CP_ROI_), outer diameter of the capsule body (d_c_) and capsule wall thickness (s_c_).

Absolute volume distribution		Individual measurements	
Capsule volume (V_CS_ROI)	603 mm^3^	Max. Length (Feret)	19.45 mm
Capsule shell volume (V_CS)	56 mm^3^	Filled Height	16.09 mm
Capsule internal volume (V_CS_InV)	548 mm^3^	Filled Volume	48.39%
Capsule void space (V_CS_Poros)	279 mm^3^	A_S, V_CS_ROI_	562.6 mm^2^
Pellet volume (V_CP_ROI)	269 mm^3^	A_S, V_CP_ROI_	1253.9 mm^2^
Pellet solid phase volume (V_CP)	201 mm^3^	d_c_	6.55 ± 0.01 mm
Pellet porosity volume (V_CP_Poros)	68 mm^3^	s_c_	112 ± 3 μm

The total internal capsule volume (V_CS_InV) is sub-divided into the pellet volume occupied by its solid phase (V_CP), large inter-pellet void space (V_CS_Poros) and intra-pellet porosity (V_CP_Poros). The pellets account for 48.39% of the internal capsule volume, less than half of V_CS_InV, corresponding to a fill height of 16.09 mm. The capsule dimensions are expressed using its maximum length (Feret) and the outer diameter of the capsule body (d_c_) which are 19.45 mm and 6.54 mm, respectively. Additionally, the capsule wall thickness (s_c_) and its surface area (A_S, V_CS_ROI_) were quantified with the associated variation in thickness across the sampled regions which are 112 ± 3 μm and 562.6 mm^2^, respectively.

**Fig. 4:**
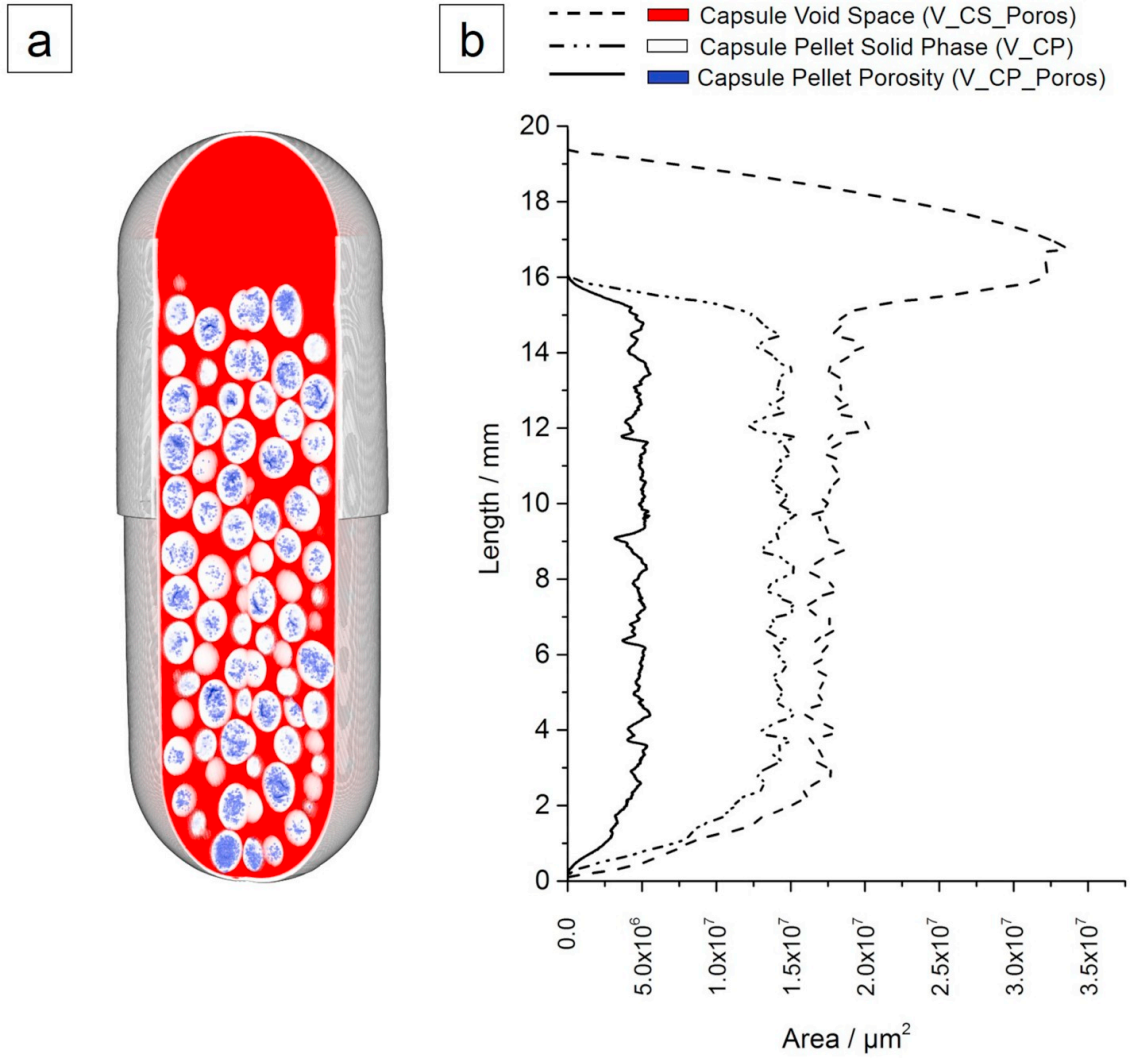
*Global* sample analysis (a) detecting internal capsule void space (V_CS_Poros, red, − – –), pellet solid phase volume (V_CP, white, − - - –) and intra-pellet porosity (V_CP_Poros, blue, −––). (b) The volume distribution along the capsule height is quantified through the detected local cross-section area in the micro-XRT image stack [coloured high-resolution figure available online]. (For interpretation of the references to colour in this figure legend, the reader is referred to the web version of this article.)

Fig. 4 shows the distribution of the pellet solid phase volume (V_CP), the pellet porosity volume (V_CP_Poros) and inter-pellet capsule void volume (V_CS_Poros) as a function of the capsule length. This data representation allows a quick assessment of the overall solid phase homogeneity within the capsule. In the distribution of V_CP and V_CS_Poros, the capsule head-space can be easily identified at a capsule height of 16.09 mm defined by a sharp decrease of V_CP and V_CP_Poros. The measured length of the capsule body is 16.78 mm and therefore, the pellets reach 95.89% of its maximum filling height.

### 3.2 Capsule - pellet segmentation

**Fig. 5:**
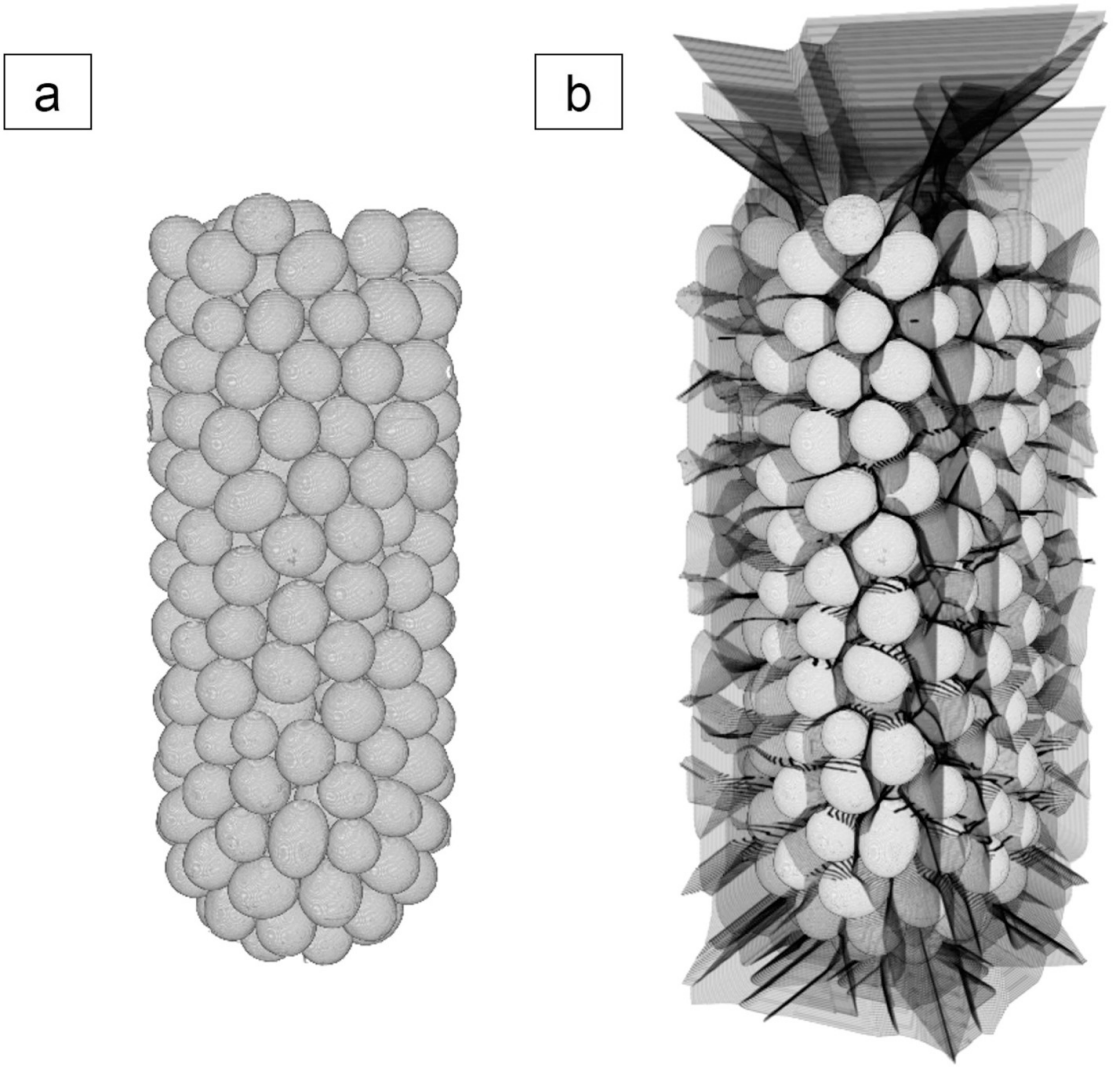
The segmentation of all “touching” pellets was achieved after applying a marker-supported watershed transformation to (a) the pellet ROI (V_CP_ROI). (b) The volume separating watershed lines are superimposed on the original image data to identify individual pellet volumes.

The extraction of structural features from individual pellets of the capsules' population requires the successful separation and isolation of all connected “touching” objects in the total pellet volume (V_CP_ROI, Fig. 5a). A marker-controlled watershed transformation was applied to enable a robust volume segmentation of V_CP_ROI. The calculated watershed lines indicate the optimised separation planes determined within the 3D image and are shown in Fig. 5b. Region markers greatly improve overall robustness against risks of over-segmentation, a common problem associated with applications of the watershed algorithm (Meyer and Beucher, 1990). The watershed algorithm is most robust for structures with large, uniform object bodies and light touching zones since the optimisation of the separation planes is based on the 3D distance map of the object ROI. The spherical pellets maximise these attributes, hence, its application is particularly effective for this sample. The separation of the pellet population in C0 resulted in 300 individual pellets. Across all capsules (DTR and DTT) the population consisted of 294 ± 9 pellets. The watershed-transformation was validated through a visual inspection of the segmented image data and the isolated single pellets.

### 3.3 Capsule - pellet characterisation

Following the successful pellet segmentation (Section 3.2), the individual pellets were subject to individual analysis aimed at extracting quantitative information using 206 structural features. The features (*n* = 206) were categorised in terms of their relation to key particle attributes of size (*n* = 61), shape (*n* = 104), surface (*n* = 12), porosity (*n* = 14) and orientation (*n* = 15). An overview of all the features is provided in Table S2 (ESI, page S2). Each feature is calculated either directly from the 3D image stack of each pellet or from three characteristic translation- and rotation-invariant IEV cross-sections as described in Section 2.3.

**Fig. 6:**
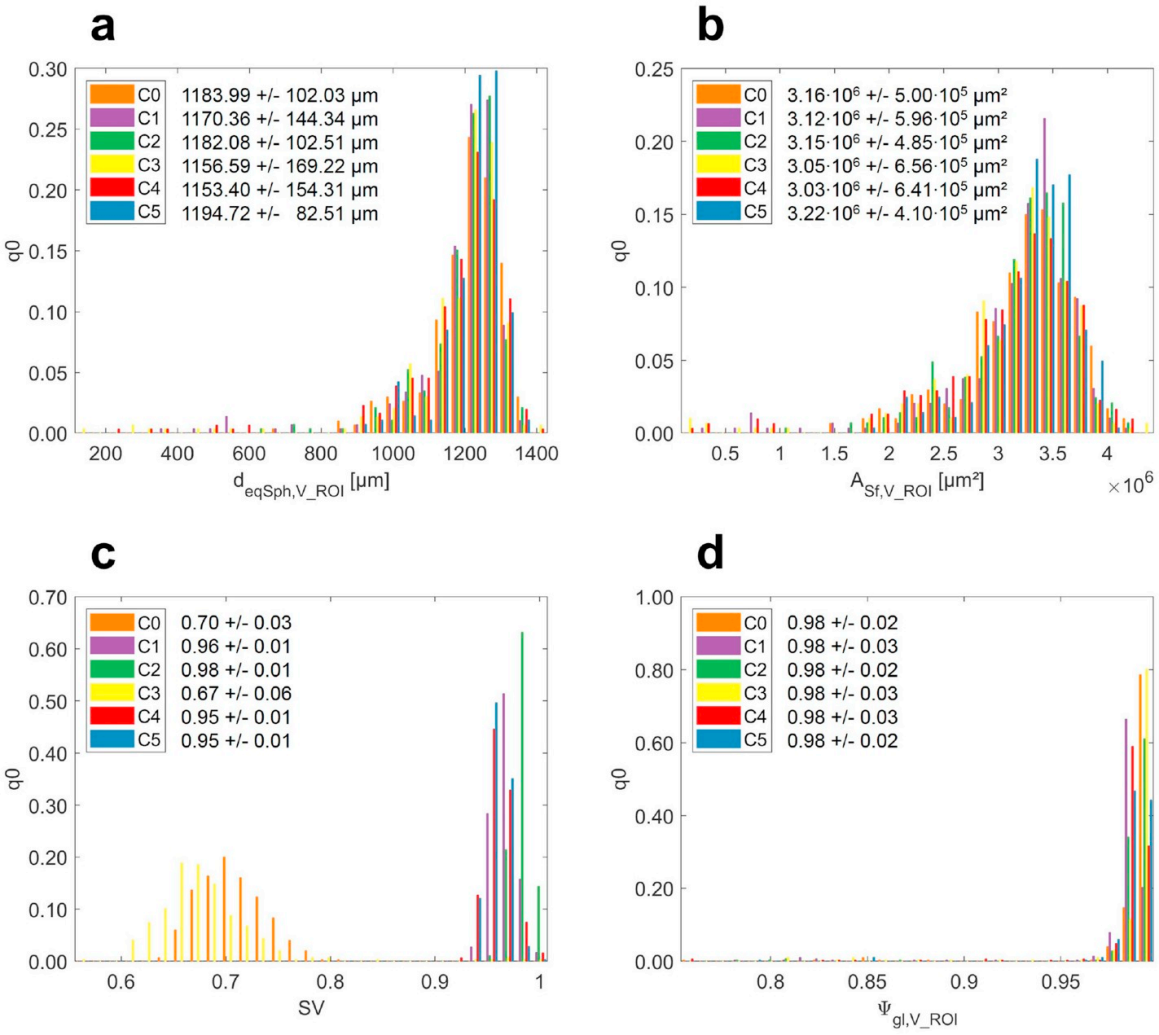
Normalised number density distributions (q0) of four selected features describing the pellet populations regarding (a) pellet size (*d*_eqSph, V_ROI_), (b) pellet surface area (A_Sf, V_ROI_), (c) pellet porosity (SV) and (d) pellet shape (Ψ_gl, V_ROI_) [coloured high-resolution figure available online].

Fig. 6 shows four selected features commonly used to describe the quality attributes of pellets from extrusion–spheronization processes that can be related to process parameters and input material attributes (Yu et al., 2014; Bryan et al., 2015): equivalent sphere diameter (*d*_eqSph, V_ROI_ ∈[90*μm*, 1408*μm*], Fig. 6a), absolute surface area (A_Sf, V_ROI_ ∈[0.02 ⋅ 10^6^*μm*^2^,4.35 ⋅ 10^6^*μm*^2^], Fig. 6b), solidity as a measure of internal porosity (SV ∈[0.55,0.99], Fig. 6c) and particle sphericity (Ψ_gl, V_ROI_ ∈[0.74,0.99], Fig. 6d). The distribution of each of the four features is visualised to provide an indication of the range of values across all pellets in the capsule. Comparing the results of the analysis, the distributions are highly consistent for *d*_eqSph, V_ROI_, A_Sf, V_ROI_, and Ψ_gl, V_ROI_ across all capsules. However, the distributions of SV in Fig. 6c show statistically significant differences (Two-sample *t*-test rejects *H*_0_ of equal means at 5% significance level). This is however a consequence of the sensitivity of the measurement to the specific micro-XRT acquisition and image analysis parameters used. The changes in SV correlate with changing data acquisition parameters between capsules with high micro-XRT image quality (slow acquisition, C0 and C3) and low micro-XRT image quality (fast acquisition, C1, C2, C4 and C5). Accelerated micro-XRT image acquisition leads to an average increase of the measured solidity (SV) from 68.71 ± 4.72% to 95.88 ± 1.48%.

The quantification of features from image data is often subject to variability in the image data quality and the selection/optimisation of image processing parameters (Yip and Aerts, 2016; Al-Sarraf et al., 2008). Therefore, a sensitivity analysis was performed to assess the robustness of extracted features within the micro-XRT analysis workflow using a customised sampling method targeting implemented algorithms for noise reduction and binarization as described in Table S3 (ESI, page S9). Changes in micro-XRT image quality were simulated using a Gaussian filter to systematically introduce image blurring. Sensitivity analysis was carried out with a dataset of five randomly selected pellets of each class (*non-broken* and *broken*) from C0. Features related to the particle porosity (V_P_Poros), the absolute particle volume (V_P) and its orientation exhibit the highest variability for different micro-XRT image data qualities and image processing parameters. This variability is linked predominantly to difficulties in the detection of micro-porosities with a local thickness below 25 μm (details are provided in Table S4, ESI, page S9). These micro-porosities contribute to 98.82 ± 1.56% of the total pellet porosity. Their critical length scale of 25 *μ*m corresponds to a minimum of 4–5 pixels in the low resolution (C1, C2, C4 and C5) and 10 pixel in the high resolution (C0 and C3) micro-XRT images, respectively. Failing to detect internal micro-porosities decreases the measured V_P_Poros and simultaneously gives an apparent rise in V_P. Small variations in V_P can further have a significant impact on the determined orientation, especially for highly isotropic objects. In total, 132 features exhibit values above a 10% variability threshold comparing the feature residuals at individual sampling points relative to the user defined ground truth (visual validation). The remaining 74 features are assumed to be robust within the employed image analysis framework. Details of the sensitivity analysis are presented qualitatively and quantitatively in Section S1.4 (ESI, page S7 - S16, Fig. S1 and Fig. S2). Depending on the objective for the micro-XRT analysis, features with a high-sensitivity to micro-XRT image quality and parameters of the image processing/analysis workflow require careful validation with complementary sample characterisation techniques. The variability of individual features depends significantly on the nature of the sample which may have to be monitored and re-evaluated for changing specimens. Here, features with a variability of more than 10% were excluded from the feature-based object classification models. Details on features removed on this basis are listed in Table S5 (ESI, page S11).

### 3.4 Capsule - pellets classification

Extracted features were utilised as part of a classification model for the automatic detection and quantification of *broken* pellets within the population. The successful implementation included a feature selection approach (Section 3.4.1). Selected features were used to build and validate SVM classification models (Section 3.4.2). The training dataset (DTR) combined pellet information from three capsules, C0 (high resolution mode), C1 and C2 (fast acquisition mode). The remaining data (C3, C4 and C5) were included in a test dataset (DTT) to validate and evaluate the model performance.

#### 3.4.1 Feature selection

74 structural pellet features were assessed using a feature selection approach to identify a subset best suited for a feature-based pellet classification model for the reliable detection of *broken* pellets within the population. The feature selection approach aims to reduce the dimensionality of the captured feature space removing redundant or noisy features. Features with a limited positive impact on the classification performance can be excluded from future micro-XRT image analysis, accelerating routine characterisation as well as improving model accuracy and robustness against overfitting. The identification and selection of predictive features is particularly important for datasets with imbalanced class membership distributions to include features that provide inter-class discrepancy and to exclude features with high intra-class noise (Chawla et al., 2004).

**Fig. 7:**
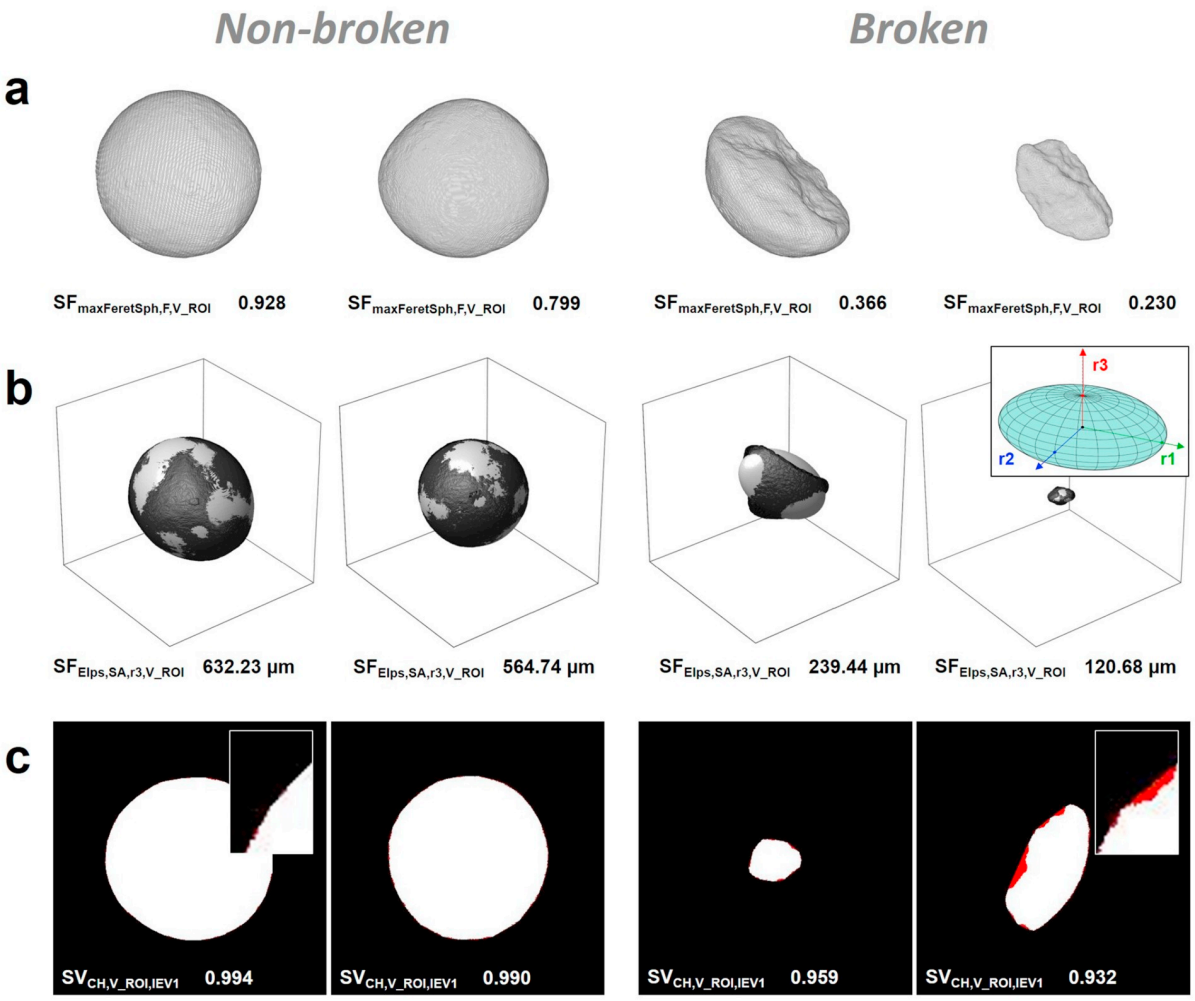
Selected features for a classification of (left) *non-broken* and (right) *broken* pellets: (a) sphericity (SF_maxFeretSph, F, V_ROI_), (b) shortest characteristic length of a fitted ellipsoid (SF_Elps, SA, r3, V_ROI_), and (c) surface roughness (SV_CH, V_ROI, IEV1_) [coloured high-resolution figure available online].

A ReliefF feature selection approach was utilised to evaluate and rank the correlation of all features with the class membership of DTR. A high correlation of individual features with the assigned *broken*/*unbroken* class membership suggests a high predictive power for the subsequent feature-based classification model. The top 5 features of the ReliefF ranking relate to shape properties. As expected for this sample with highly spherical pellets, the highest ranking feature describes the overall pellet sphericity comparing the pellet volume to a sphere with equal maximum Feret diameter (SF_maxFeretSph, F, V_ROI_, rank 1, Fig. 7a). Additional high-ranked features from other structure-related categories are quantitative information on the pellets' size (SF_Elps, SA, r3, V_ROI_, rank 6, Fig. 7b) and surface attributes (SV_CH, V_ROI, IEV1_, rank 7, Fig. 7c). Interestingly, SV_CH, V_ROI, IEV1_ is closely related to SV_CH, V, IEV1_ which captures particle porosity information using a 2D Convex-Hull. Applied to V_ROI, the same algorithm quantifies concave areas on the object surface (Doerr et al., 2018). Therefore, SV_CH, V_ROI, IEV1_ can be regarded as a measure of surface roughness. SF_Elps, SA, r3, V_ROI_ is linked to the pellet size and is an absolute measure of the shortest characteristic length of a surface-fitted ellipsoid to V_ROI. In contrast, the other remaining characteristic lengths of this ellipsoid, SF_Elps, SA, r1, V_ROI_ and SF_Elps, SA, r2, V_ROI_, are at position 39 and 36 of the ReliefF ranking, respectively. The high performance of SF_Elps, SA, r3, V_ROI_ not only relates to the expected smaller absolute sizes of the *broken* pellets, but also to its ability to capture specific shape-related aspects with an increasing deviation between SF_Elps, SA, r1, V_ROI_ and SF_Elps, SA, r3, V_ROI_ for non-spherical objects. The combination of both, aspects of size and shape, is beneficial to distinguish between *broken* and *non-broken* pellets. The results of the ReliefF ranking align well with calculated *p*-Values for DTR, where 72 of the 74 features show a statistically significant deviation between the feature distribution of *non-broken* and *broken* pellets (Two-sample *t*-test rejects *H*_0_ of equal means at 5% significance level). F-scores are an alternative approach to select features for SVM classifiers (Chen and Lin, 2006) and were compared to validate the feature selection approach. They show a similar feature ranking, however, fail to address important feature-feature dependencies. The full ReliefF ranking of the 74 investigated pellet features and their calculated ReliefF weights, p-Values and F-scores are listed in Table S6 (ESI, page S16).

Three features, SF_maxFeretSph, F, V_ROI_, SF_Elps, SA, r3, V_ROI_ and SV_CH, V_ROI, IEV1_, from distinct structure-related categories describing shape, size and surface properties were included in the selected feature subset for a pellet classification model. Each feature is presented in Fig. 7 a-c with representative examples of its extreme values (first and fourth pellet from the left) as well as an example at the population mean of each, *non-broken* and *broken* pellets (second and third pellet from the left). Scatter-plots of all feature combinations are provided in Fig. S3 (ESI, page S20). SF_maxFeretSph, F, V_ROI_ and SF_Elps, SA, r3, V_ROI_ have both a broad distribution in comparison to SV_CH, V_ROI, IEV1_. The narrow distribution of SV_CH, V_ROI, IEV1_ correlates to the high convexity of the spherical shape and aligns with visual inspections of individual pellets which exhibit highly smoothed surfaces, even for *broken* pellets. This could indicate that pellet breakage occurs predominantly during the spheronization process itself, where freshly created *broken* pellet pieces experience high attrition and smoothing of their edges. The selected feature subset from distinct structure-related categories aims to increase the robustness of the classification model against potential variability in individual structure-related categories with risks of high feature correlation.

#### 3.4.2 Support Vector machine for binary classification of formulated pellets

**Fig. 8:**
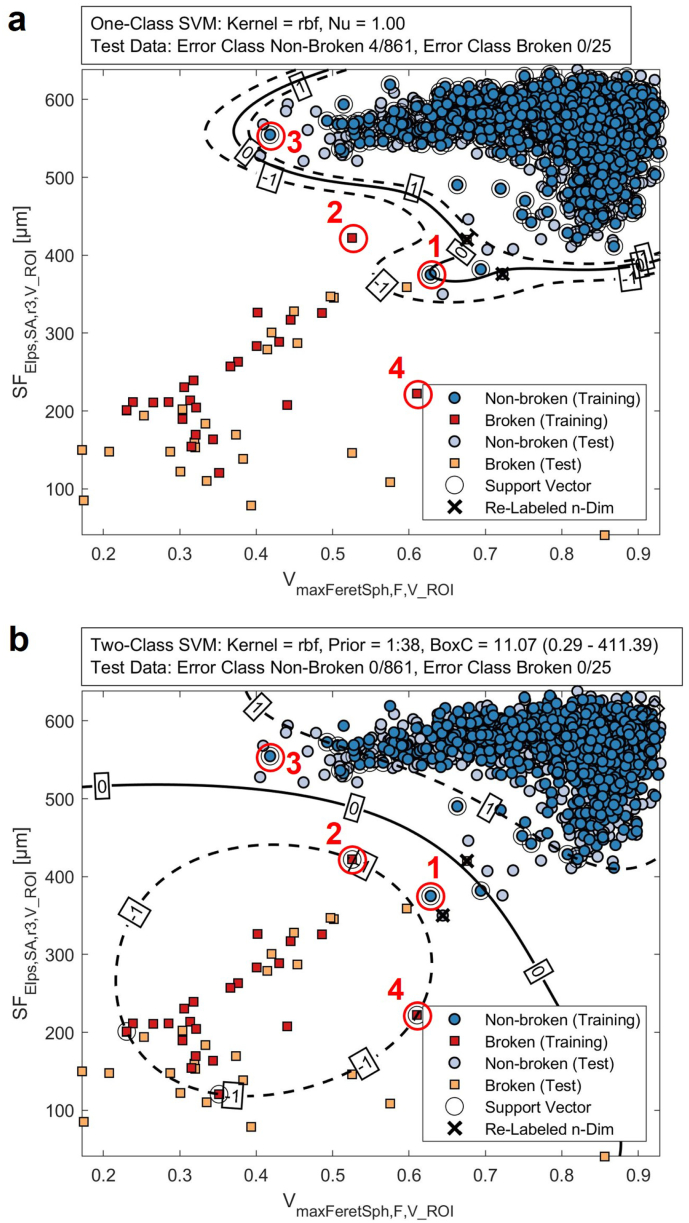
SVM models with RBF kernels for the feature-based classification of 886 pellets in DTT (−––– OSH: SVM score 0, − – – OSH margin: SVM score ∈[−1, 1]). (a) OC-SVM generates a tight OSH around the feature point-cloud of all *non-broken* pellets with a high sensitivity towards data outliers. The OC-SVM has an accuracy of 99.55% (precision *broken* pellets: 86.20%). (b) TC-SVM successfully classified all pellets in DTT. (×) Individual objects were re-classified after expanding the feature space including SV_CH, V_ROI, IEV1_. (Red circles 1-4) Selected TC-SVM support vectors are compared in details in Fig. 9 [coloured high-resolution figure available online].

The number of broken pellets is expected to be highly under-represented in the overall pellet population with an expected probability of less than 5%. In case of DTR the *broken* pellets account for 2.33% (C0, *n* = 7), 3.77% (C1, *n* = 11) and 1.75% (C2, *n* = 5) of the total pellet population, respectively. The combined training dataset (DTR) has a class imbalance of 1:38 for *broken*:*non-broken* pellets. A One-Class SVM (OC-SVM) model and a Two-Class SVM (TC-SVM) model are compared in the following to assess their performance addressing risks of a high class imbalance. The results are shown in Fig. 8a and b, respectively.

OC-SVMs generate models with highly sensitive decision surfaces which are often used for outlier-detection, where only very limited or no training data for outliers, the minority class, are available (Perdisci et al., 2006). The trained OC-SVM model is shown in Fig. 8a. The OC-SVM creates a tight decision boundary around the feature data of the *non-broken* pellet population of the pre-classified DTR data (*n* = 854, dark blue circles). Population outliers in this model are related to observations of *broken* pellets (*n* = 23, red squares). Using a RBF kernel, the decision boundary can be improved to capture non-linear feature distributions. Expanding the feature space to include information on the pellet surface roughness (SV_CH, V_ROI, IEV1_) leads to a re-classification of 5 *non-broken* pellets (Fig. 8a, × *Re-Labelled n-Dim*), slightly improving the OC-SVM model with a total classification accuracy of 99.55% (precision *broken* pellets: 86.20%) assessed using test data of a population of 886 pellets in DTT. The high sensitivity of the OC-SVM model decision surface leads to a misclassification of 4 *non-broken* pellets as *broken*, which corresponds to 0.46% of the 861 *non-broken* pellets in the DTT. In contrast, all *broken* pellets were correctly identified. Increasing the training dataset over time after reviewing and including observations of pellets in the OSH margin region can further improve the classification accuracy for *non-broken* pellets, but might shift the decision boundary towards the *broken* pellet population.

Alternatively, the training data (DTR) can be used as part of a TC-SVM to create a OSH considering feature information from both classes of *broken* and *non-broken* pellets. The trained TC-SVM model with RBF kernel is shown in Fig. 8b. Prior probabilities were selected proportional to the known class membership distribution of DTR (1:38, *broken*:*non-broken*) specifically penalizing the misclassification of *broken* pellets and shifting the OSH towards the majority class. The TC-SVM models' hyperparameter optimisation yielded changing values for the applied box constraints (BoxC) of both classes with high regularization for the majority *non-broken* pellet class (BoxC = 0.29) and low regularization with hard margins for the minority *broken* pellet class (BoxC = 411.39), indicating significant differences in the model capability to generalize observations of both classes. The TC-SVM model correctly classified all 886 pellets in the test set (DTT) using feature data of SF_maxFeretSph, F, V_ROI_, SF_Elps, SA, r3, V_ROI_ and SV_CH, V_ROI, IEV1_. In comparison to the OC-SVM model, the classification accuracy is further improved, however, the TC-SVM model increases risks for the misclassification of *broken* pellets, significantly extending the OSH margins for *non-broken* pellets. Especially for areas of the feature space represented by only few or no observations in the DTR, the TC-SVM model could fail to detect population anomalies with highly unusual feature combinations. These could result from significant changes in the manufacturing process and are expected to be located in the OSH margin. Similar to the OC-SVM model, individual cases in proximity to the OSH margin can be reviewed in order to identify potential cases of pellet misclassification and further improve the model robustness over time. For the TC-SVM model the number of observations in the OSH margin increases to 40 pellets (*non-broken* 30, *broken* 10) compared to 4 pellets (*non-broken* 4, *broken* 0) for the OC-SVM model.

**Fig. 9:**
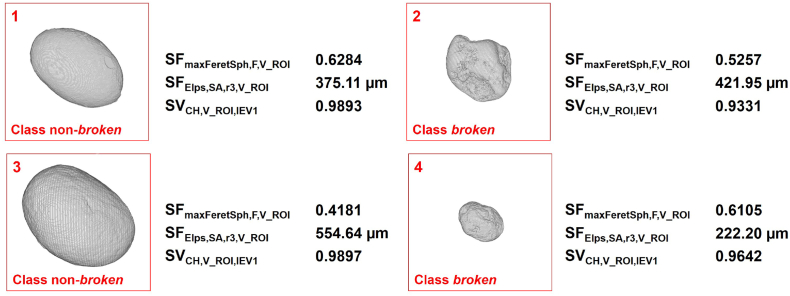
Individual (1,3) *non-broken* and (2,4) *broken* pellets with feature combinations associated to support vectors of the TC-SVM classification model defining the OSH margin. The examples visualise the importance of a multi-dimensional feature space targeting distinct pellet properties related to shape, size and surface characteristics. Large *broken* pellets (pellet 2, high SF_Elps, SA, r3, V_ROI_) can be distinguished by their surface characteristics (SV_CH, V_ROI, IEV1_). Small, rounded pellet pieces (pellet 4) can be identified using SF_Elps, SA, r3, V_ROI_ [coloured high-resolution figure available online].

Selected pellets acting as support vectors of the TC-SVM model are shown with their corresponding feature combinations in Fig. 9. These pellets contain feature combinations located in proximity of the OSH. The direct comparison supports the selection of features from distinct structure-related categories to capture the full spread of the *broken* pellet population. Pellet 2 has a SF_Elps, SA, r3, V_ROI_ of 421.95 μm which is larger than the smallest *non-broken* pellet in DTR (Pellet 1, 375.11 μm). However, Pellet 2 can be easily distinguished using information on surface roughness (SV_CH, V_ROI, IEV1_), where the population of *non-broken* pellets exhibits a narrow distribution of 0.989 ± 0.002. In contrast, Pellet 3 has a low sphericity quantified using SF_maxFeretSph, F, V_ROI_, but a large absolute size expressed in SF_Elps, SA, r3, V_ROI_. In general, smaller *broken* pellets tend to exhibit a wider distribution of shape- and surface-related features (i.e. Pellet 4). Here, SF_Elps, SA, r3, V_ROI_ as an absolute measurement of the pellet size ensures a robust classification.

The high accuracy and precision of the SVM models suggest a strong performance of the selected feature space to solve this classification problem aiming to identify *broken* pellets in each capsule. The successful quantitative characterisation of the population of pellets across all measured capsules can be used to evaluate the sample against product specifications for quality control or to assess the impact of changing formulation and/or process parameters. Additionally, the non-destructive nature of the micro-XRT analysis allows a correlation of extracted information to the sample's performance data which may explain and ultimately predict final product performance such as dissolution where particle breakage may have an effect. The presented micro-XRT analysis approach has the potential to be translated to other pharmaceutical systems such as crystallisation, spherical agglomeration, granulation and tableting. Feature selection and classification models can be adapted to target changing research objectives. The extraction of quantitative information from these systems with complex multi-dimensional structural properties can help to improve the understanding of product transformations within individual unit operations to optimise pharmaceutical manufacturing processes and accelerate process development. In combination with quality controls for managing extensive experimental data, ML strategies can be utilised to identify patterns and inference in the product characteristics.

## 4 Conclusions

Micro-XRT was successfully employed to characterise a solid pharmaceutical multi-particulate product quantifying structural attributes of the entire sample and of its primary particles. The implemented algorithms to reliably extract features of the primary particles were essential to allow an in-depth statistical evaluation of the population and were utilised in the first instance to assess pellet uniformity. Feature robustness was further tested against variations in image quality and image processing parameters showing significant feature-dependent deviations, hence promoting the importance of a sensitivity analysis for applications of a quantitative micro-XRT characterisation. The combination of image analysis with feature selection and ML methodologies was essential for the recognition of underlying patterns in these high dimensional datasets with complex structure-related feature combinations. Here, it allowed the automatic detection of all 25 *broken* pellets within a test dataset of 886 pellets at a minimum accuracy of 99.55% and a minimum precision for the classification of *broken* pellets of 86.20%. The application of this systematic characterisation workflow combining quantitative micro-XRT analysis with ML models shows promising performance as a novel approach for an automated analysis of micro-XRT image data. These analysis frameworks are invaluable across a wide range of pharmaceutical multi-particle systems, and only restricted by limitations of the micro-XRT image acquisition system. Quantitative information on structural particle properties have direct applicability in quality control and can be utilised to inform product and process development. The non-destructive nature of this characterisation method permits the additional assessment of the product performance to gain valuable insights into structure-performance relationships for pharmaceutical systems. Future work will focus on the translation of the demonstrated capabilities to a wider range of pharmaceutical solid products.

## Declaration of Competing Interest

The authors declare that they have no known competing financial interests or personal relationships that could have appeared to influence the work reported in this paper.

## Appendix A Supplementary data

Supplementary material Image 1

Supplementary video Image 2
