# Supplementary material for: A micro-XRT image analysis and machine learning methodology for the characterisation of multi-particulate capsule formulations
Source: Int J Pharm X. 2020 Jan 15;2:100041. doi: 10.1016/j.ijpx.2020.100041 (PMC6997304; doi:10.1016/j.ijpx.2020.100041)
Supplement: Supplementary file 1 — Supplementary material [file mmc1.pdf]

Electronic Supplementary Material

Title: A micro-XRT Image Analysis and Machine Learning Methodology for the Characterisation of Multi-Particulate Capsule Formulation

Authors: Frederik J. S. Doerr and Alastair J. Florence

Journal: International Journal of Pharmaceutics

Accepted Date: 18 Nov. 2019

DOI: https://doi.org/10.1016/j.ijpx.2020.100041

DATA DOI: https://doi.org/10.15129/e5d22969-77d4-46a8-83b8-818b50d8ff45

S1.1. Micro-XRT Data Acquisition Details

Table S1: Micro-XRT data acquisition settings for scanned ibuprofen capsules. C0 and C3 were collected aiming for high image data quality. C1, C2, C4 and C5 were collected with accelerated scanning settings for fast image acquisition.

**Sample**	Source Voltage / Current	Image Pixelsize	Frame Averaging / Step Size	Scan Time
C0	40 kV/380 μA	2.5 μm	3/0.2°	12h:08min
C1	40 kV/400 μA	5.0 μm	3/0.2°	01h:57min
C2	40 kV/400 μA	6.0 μm	3/0.2°	02h:00m
C3	40 kV/360 μA	2.5 μm	3/0.2°	16h:24min
C4	40 kV/400 μA	5.0 μm	3/0.2°	02h:01min
C5	40 kV/400 μA	5.0 μm	3/0.2°	01h:58min

S1.2. *Voxel-based arithmetic Operations*

The internal capsule volume (V_CS_InV) was calculated from the capsule shell (V_CS) and its ROI (V_CS_ROI, Equation S1). To differentiate between inter- and intra-object background volumes a pellet ROI was defined (V_CP_ROI) as previously described for single particles [1]. V_CP_ROI was subsequently subtracted from V_CS_InV to yield the capsule void space (V_CS_Poros, Equation S2). The total pellet porosity (V_CP_Poros) is given after a subtraction of the pellets' solid phase (V_CP) from V_CP_ROI using Equation S3. The distribution of both can be quantified for the full sample and locally within the capsule image space.

$$\text{V\_CS\_InV} = \text{V\_CS\_ROI} - \text{V\_CS} \tag{S1}$$

$$\text{V\_CS\_Poros} = \text{V\_CS\_InV} - \text{V\_CP\_ROI} \tag{S2}$$

$$\text{V\_CP\_Poros} = \text{V\_CP\_ROI} - \text{V\_CP} \tag{S3}$$

S1.3. *Micro-XRT Image Analysis Features*

Table S2: Overview of structural features extracted from each formulated pellet after volume segmentation. A total 206 features were used for the evaluation of the particle population. [a] CMAC, [b] MATLAB Image Processing Toolbox, [c] literature/Mathworks File Exchange

Size Features	
Object Volume (V)[b]	Absolute size of all pixels/voxels of the object volume (translation- and rotation-invariant, 2D/3D, $\in \mathbb{R}^+$).
Eqv. Sphere Diameter (d_{eqSph})[b]	Equivalent spherical diameter of the object volume (translation- and rotation-invariant, 2D/3D, $\in \mathbb{R}^+$).

Bounding Box (V_{BB}, $\mathrm{SF}_{\mathrm{BB}}$)[b]	Size and characteristic lengths of the minimum bounding box incorporating the object volume (translation-invariant, 2D/3D, $\in \mathbb{R}^+$).
Max Feret Diameter (d_{maxFeret})[a]	Maximum distance between two points on the object surface (translation- and rotation-invariant, 2D/3D, $\in \mathbb{R}^+$).
Mean Breadth Diameter (d_{Bdth})[c]	Mean breadth of the object orthogonal to the object length (translation- and rotation-invariant, 2D/3D, $\in \mathbb{R}^+$)
Ellipsoid Fitting ($\mathrm{SF}_{\mathrm{Elps,IM}}$)[b]	Characteristic lengths of a fitted ellipsoid/ellipse with equivalent second central image moments (translation- and rotation-invariant, 2D/3D, $\in \mathbb{R}^+$).
Ellipsoid Fitting ($\mathrm{SF}_{\mathrm{Elps,SA}}$)[c]	Characteristic lengths of a surface-fitted ellipsoid/ellipse (translation- and rotation-invariant, 2D/3D, $\in \mathbb{R}^+$) [2].
Ellipsoid Fitting ($\mathrm{SF}_{\mathrm{Elps,minB}}$)[c]	Characteristic lengths of the minimum bounding ellipsoid/ellipse incorporating the object volume (translation- and rotation-invariant, 2D/3D, $\in \mathbb{R}^+$) [3].
Sphere Fitting ($\mathrm{SF}_{\mathrm{Sph,minB}}$)[a,c]	Radius of the minimum bounding circle/sphere incorporating the object volume (translation- and rotation-invariant, 2D/3D, $\in \mathbb{R}^+$) [3].
Half Sphere Fitting ($\mathrm{SF}_{\mathrm{HalfSph,minB}}$)[c]	Characteristic lengths of the minimum bounding half circle/sphere incorporating the object volume (translation- and rotation-invariant, 2D, $\in \mathbb{R}^+$) [3].
Rectangle Fitting ($\mathrm{SF}_{\mathrm{Rect,minB,l}}$)[c]	Characteristic lengths of the minimum bounding rectangle incorporating the object volume (translation- and rotation-invariant, 2D, $\in \mathbb{R}^+$) [3].

Triangle Fitting ($SF_{Tri,minB}$)[c]	Characteristic lengths of the minimum bounding triangle incorporating the object volume (translation- and rotation-invariant, 2D, $\in \mathbb{R}^+$) [3].
Shape Features	
Eccentricity (ECC)[b]	Ratio of the distance between the foci of the ellipse and its major axis length (translation- and rotation-invariant, 2D, $\in [1, \infty] \subset \mathbb{R}^+$).
Aspect Ratio ($AR_{Elps,IM}$)[a]	Ratio between longest and shortest characteristic length of a fitted ellipsoid with equivalent second central image moments (translation- and rotation-invariant, 2D/3D, $\in [1, \infty] \subset \mathbb{R}^+$).
Sphericity, global (Ψ_{gl})[a]	Sphericity index calculated as the ratio of the detected object volume to its surface (translation- and rotation-invariant, 2D/3D, $\in [0, 1] \subset \mathbb{R}^+$) [4].
Local Roundness (Ψ_{lR})[c]	Detection of local roundness including local roundness ratios and smallest local surface curvature ratio (translation- and rotation-invariant, 2D, $\in [0, 1] \subset \mathbb{R}^+$) [5]
Sphere Fitting ($SF_{maxFeretSph,F}$)[a]	Goodness-of-fit for circle/sphere with equivalent maximum Feret diameter (translation- and rotation-invariant, 2D, $\in [0, 1] \subset \mathbb{R}^+$).
Ellipsoid Fitting ($SF_{Elps,SA,F}$)[c]	Goodness-of-fit for surface-fitted ellipsoid/ellipse. Linear least squares optimisation (translation- and rotation-invariant, 2D/3D, $\in \mathbb{R}^+$) [2].
Ellipsoid Fitting ($SF_{Elps,minB,F}$)[c]	Goodness-of-fit for minimum bounding ellipsoid/ellipse incorporating the object volume (translation- and rotation-invariant, 2D/3D, $\in [0, 1] \subset \mathbb{R}^+$) [3].

Sphere Fitting ($SF_{Sph,minB,F}$)[c]	Goodness-of-fit for minimum bounding circle/sphere incorporating the object volume (translation- and rotation-invariant, 2D, $\in [0,1] \subset \mathbb{R}^+$) [3].
Half Sphere Fitting ($SF_{HalfSph,minB,F}$)[c]	Goodness-of-fit for minimum bounding half circle/sphere incorporating the object volume (translation- and rotation-invariant, 2D, $\in [0,1] \subset \mathbb{R}^+$) [3].
Rectangle Fitting ($SF_{Rect,minB,F}$)[c]	Goodness-of-fit for minimum bounding rectangle incorporating the object volume (translation- and rotation-invariant, 2D, $\in [0,1] \subset \mathbb{R}^+$) [3].
Parallelogram Fitting ($SF_{Pg,minB,F}$)[c]	Goodness-of-fit for minimum bounding parallelogram incorporating the object volume (translation- and rotation-invariant, 2D, $\in [0,1] \subset \mathbb{R}^+$) [3].
Triangle Fitting ($SF_{Tri,minB,F}$)[c]	Goodness-of-fit for minimum bounding triangle incorporating the object volume (translation- and rotation-invariant, 2D, $\in [0,1] \subset \mathbb{R}^+$) [3].
Zernike (Z_A)[c]	Amplitude of complex zernike moment (translation- and rotation-invariant, 2D, $\in [0,1] \subset \mathbb{R}^+$) [6].
Surface Features	
Surface Area (A_{Sf})[b]	Absolute area of the object boundary region (translation- and rotation-invariant, 2D/3D, $\in \mathbb{R}^+$).
Surface Roughness (SV_{CH,V_ROI})[a,b]	Ratio between object ROI volume and the convexhull volume (translation- and rotation-invariant, 2D/3D, $\in [0,1] \subset \mathbb{R}^+$).
Porosity Features	

Porosity<br>$(\mathrm{P})^a$	Absolute area/volume of the object's open, closed or total porosity (translation- and rotation-invariant, 2D/3D, $\in \mathbb{R}^+$).
Convex-hull Solidity<br>$(\mathrm{SV}_{\mathrm{CH,V}})^{a,b}$	Ratio between object volume and its convex-hull volume (translation- and rotation-invariant, 2D/3D, $\in [0,1] \subset \mathbb{R}^+$).
Solidity<br>$(\mathrm{SV})^a$	Ratio between object volume and its ROI volume (translation- and rotation-invariant, 2D/3D, $\in [0,1] \subset \mathbb{R}^+$).
Extend<br>$(\mathrm{Ex})^b$	Ratio between object volume and minimum bounding box volume (translation-invariant, 2D/3D, $\in [0,1] \subset \mathbb{R}^+$).
Orientation/Location Features	
Centroid<br>$(x_c, y_c, z_c)^b$	Coordinates of the regional centre of mass from first image moment (rotation-invariant, 2D/3D, $\in \mathbb{R}^+$).
Euler angles<br>$(\alpha_E, \beta_E, \gamma_E)^b$	Euler angle between the characteristic object eigenvectors determined from the second centralised image moments (translation-invariant, 3D, $\in [-180, 180] \subset \mathbb{R}$).
Orientation<br>$(\alpha)^b$	Angle between the x-axis and the major axis of a fitted ellipse with equivalent second central image moments (translation-invariant, 2D, $\in [-180, 180] \subset \mathbb{R}$).
Zernike<br>$(Z_\mathrm{P})^c$	Phase angle of Zernike moment (translation-invariant, 2D, $\in [-180, 180] \subset \mathbb{R}$) [6].

S1.4. Micro-XRT Sensitivity Analysis Details

The impact of changing micro-XRT image quality and image processing parameters on the quantified structural features was investigated during a sensitivity analysis. Sampled conditions of changing micro-XRT image quality and image processing parameters are described in Table S3 (ESI, page S9). The results are presented qualitatively and quantitatively in Fig. S1 (ESI, page S8) and Fig. S2 (ESI, page S10), respectively. Features with the highest variability are related to the particle porosity, which further impacts features describing the object's volume and orientation. The changes in the pellet porosity were further assessed using an approach for structural thickness calculation (CTAn v 1.16.4.1, Bruker). The local thickness of the pellet porosity (V_P_Poros) is compared in Table S4 as volume density distributions normalised to the user defined ground truth (SA_01). The total porosity volumes change significantly with up to 92.44 % difference between SA_06 and SA_03. The smaller porosity fractions between 5 μm and 25 μm are particularly sensitive to changing micro-XRT image qualities and image processing parameters indicating a size dependent sensitivity. For SA_01 (user optimised parameters), 99.07 % of the total porosity volume are part of the 5 μm and 25 μm size range emphasising the overall impact of these two size fractions to the total porosity volume.

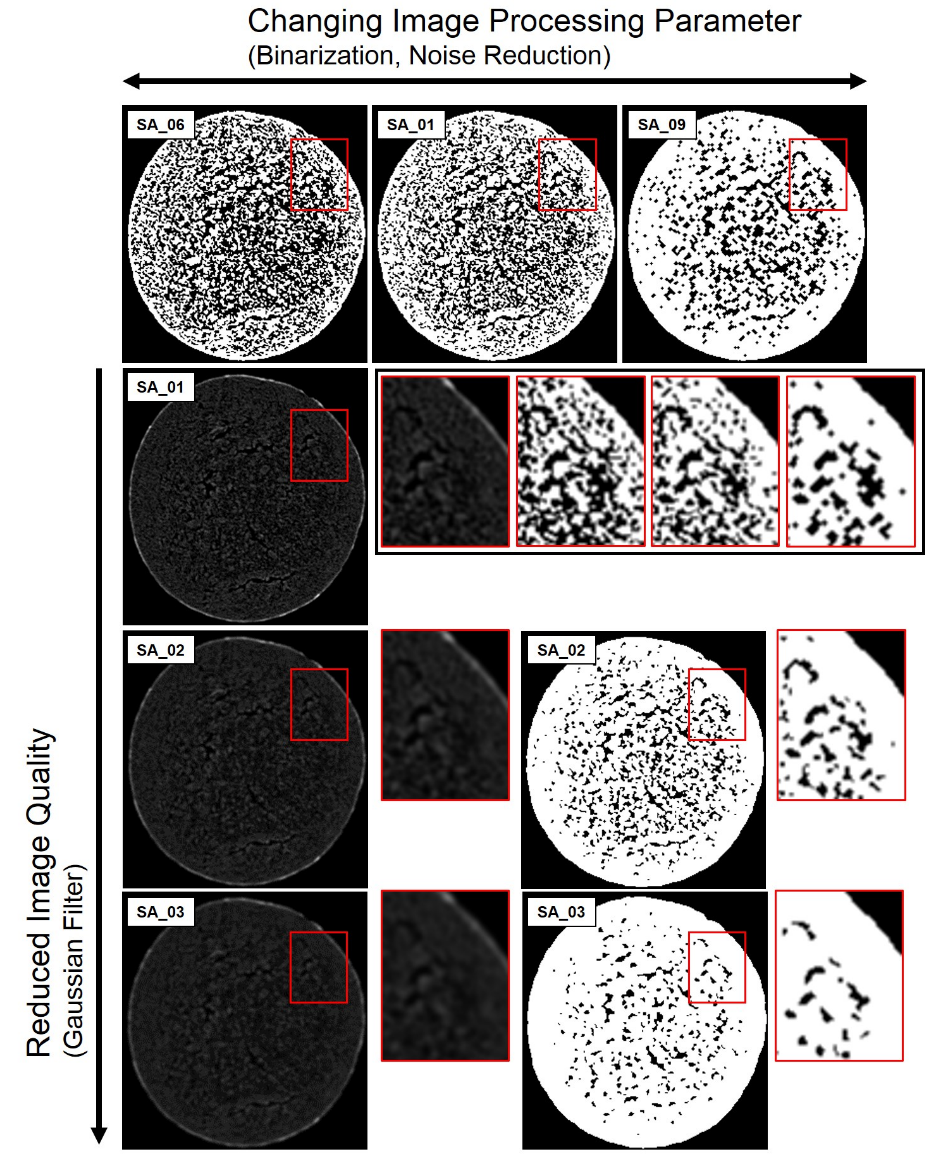

Figure S1: Qualitative visualisation of the impact of the collected micro-XRT image quality and critical image processing parameters on the binarized object volume. The images suggest a strong correlation between the detected internal object porosity and the collected micro-XRT image data quality as well as selected processing parameters.

Table S3: Overview of sampling points for a sensitivity analysis systematically assessing the impact of changes in the micro-XRT image quality and image processing parameters.

**ID**	**Info**
SA_01	*Optimised parameter (ground truth, user validated)*
SA_02	Reduced micro-XRT image quality (gaussian filter, $\sigma = 0.8$)
SA_03	Reduced micro-XRT image quality (gaussian filter, $\sigma = 1.2$)
SA_04	Ridler-Calvard (alternative image thresholding method)
SA_05	Image threshold value -1.6% of SA_01
SA_06	Image threshold value +1.6% of SA_01
SA_07	No V_ROI noise reduction
SA_08	High V_ROI noise reduction
SA_09	Medium V noise reduction
SA_10	High V noise reduction

Table S4: Relative changes in the porosity volume distribution for selected samples during a sensitivity analysis and the contribution related to the local thickness. The smallest porosity fraction between 5.0 - < 15.0 μm is particularly affected by changes in the micro-XRT image quality and image processing parameters.

Range [μm]	SA_01	SA_02	SA_03	SA_06	SA_09
5.0 - < 15.0	90.12%	30.57%	5.22%	110.42%	31.91%
15.0 - < 25.0	8.95%	3.62%	1.48%	18.40%	8.66%
25.0 - < 35.0	0.53%	0.40%	0.47%	0.61%	0.59%
35.0 - < 45.0	0.24%	0.15%	0.23%	0.27%	0.24%
45.0 - < 55.0	0.12%	0.09%	0.12%	0.13%	0.13%
55.0 - < 65.0	0.02%	0.03%	0.05%	0.01%	0.02%
65.0 - < 75.0	0.02%	0.00%	0.00%	0.03%	0.02%
**Sum**	**100.00%**	**34.86%**	**7.56%**	**129.86%**	**41.57%**

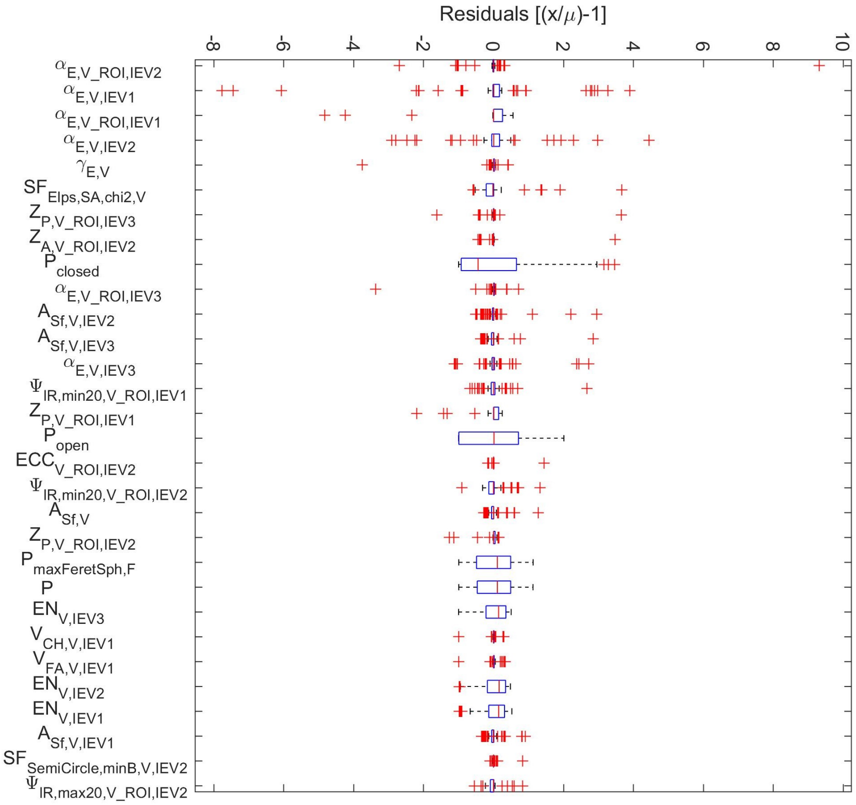

Figure S2: Box plots of the top 30 features with the highest variability during a quantitative sensitivity analysis of micro-XRT image quality and critical image processing parameters on the extracted object features (x = observation, μ = feature mean). The sensitivity analysis identified features linked to the pellet porosity and orientation with the largest dependency on collected data quality and selected processing parameters.

Table S5: Sensitivity analysis ranking of 132 structural features exhibiting values above a 10% variability threshold comparing the feature residuals of individual sampling points to the user defined ground truth. These features were excluded from the feature selection approach for pellet classification.

Feature	Rank	Residuals (X/μ-1)			Category
		Median	Max	Min	
$\alpha_{E,V\_ROI,IEV2}$	1	0	9.309	-2.7	Orientation
$\alpha_{E,V,IEV1}$	2	0.006	3.895	-7.761	Orientation
$\alpha_{E,V\_ROI,IEV1}$	3	0	0.556	-4.827	Orientation
$\alpha_{E,V,IEV2}$	4	0.004	4.442	-2.912	Orientation
$\gamma_{E,V}$	5	0.001	0.426	-3.76	Orientation
$SF_{Elps,SA,chi2,V}$	6	-0.018	3.665	-0.59	Shape
$Z_{P,V\_ROI,IEV3}$	7	0	3.648	-1.625	Orientation
$Z_{A,V\_ROI,IEV2}$	8	0	3.482	-0.449	Size
P_{closed}	9	-0.443	3.46	-1	Poros
$\alpha_{E,V\_ROI,IEV3}$	10	0.001	0.714	-3.373	Orientation
$A_{Sf,V,IEV2}$	11	-0.001	2.954	-0.508	Surface
$A_{Sf,V,IEV3}$	12	-0.005	2.85	-0.375	Surface
$\alpha_{E,V,IEV3}$	13	0.002	2.72	-1.125	Orientation
$\Psi_{lR,min20,V\_ROI,IEV1}$	14	0.002	2.673	-0.678	Shape
$Z_{P,V\_ROI,IEV1}$	15	0.007	0.248	-2.196	Orientation
P_{open}	16	0.013	2.01	-1	Poros
$ECC_{V\_ROI,IEV2}$	17	0.001	1.441	-0.166	Shape
$\Psi_{lR,min20,V\_ROI,IEV2}$	18	-0.003	1.332	-0.911	Shape
$A_{Sf,V}$	19	-0.001	1.276	-0.278	Surface
$Z_{P,V\_ROI,IEV2}$	20	0.001	0.148	-1.263	Orientation

$P_{maxFeretSph,F}$	21	0.107	1.131	-0.996	Poros
P	22	0.107	1.128	-0.996	Poros
$EN_{V,IEV3}$	23	0.144	0.503	-1	Poros
$V_{CH,V,IEV1}$	24	-0.001	0.299	-1	Shape
$V_{FA,V,IEV1}$	25	-0.001	0.334	-1	Poros
$EN_{V,IEV2}$	26	0.159	0.481	-0.99	Poros
$EN_{V,IEV1}$	27	0.147	0.523	-0.989	Poros
$A_{Sf,V,IEV1}$	28	-0.005	0.906	-0.344	Surface
$SF_{SemiSph,minB,V,IEV2}$	29	-0.003	0.829	-0.108	Shape
$\Psi_{lR,max20,V\_ROI,IEV2}$	30	-0.002	0.827	-0.545	Shape
$\Psi_{lR,max20,V\_ROI,IEV1}$	31	0	0.813	-0.473	Shape
$V_{V,IEV2}$	32	-0.052	0.774	-0.271	Size
$\alpha_{E,V}$	33	0.001	0.497	-0.744	Orientation
$\Psi_{lR,V\_ROI,IEV2}$	34	0.001	0.092	-0.654	Shape
$ECC_{V,IEV2}$	35	0.003	0.118	-0.63	Shape
$\Psi_{lR,min20,V\_ROI,IEV3}$	36	0	0.626	-0.367	Shape
$d_{breadth,V\_ROI}$	37	0.001	0.128	-0.567	Size
$\Psi_{lR,V\_ROI,IEV1}$	38	0.001	0.557	-0.284	Shape
$EX_{V,IEV2}$	39	-0.021	0.544	-0.365	Shape
$SF_{Tri,minB,V,IEV2}$	40	-0.002	0.524	-0.071	Shape
$SF_{SemiSph,minB,V\_ROI,IEV1}$	41	-0.002	0.506	-0.061	Shape
$SF_{SemiSph,minB,V\_ROI,IEV2}$	42	-0.002	0.062	-0.49	Shape
$SF_{Sph,minB,F,V,IEV2}$	43	0.001	0.477	-0.323	Shape
$SF_{PolyArea,V,IEV2}$	44	-0.001	0.463	-0.327	Shape
$V_{FA,V,IEV2}$	45	-0.001	0.448	-0.269	Poros
$SF_{Sph,minB,r,V}$	46	0.004	0.08	-0.44	Size
$EX_{V,IEV3}$	47	-0.031	0.379	-0.359	Shape

$SV_{V,IEV2}$	48	-0.027	0.376	-0.334	Poros
$SF_{Elps,IM,dMin,V,IEV2}$	49	0.001	0.375	-0.054	Size
$\Psi_{lR,V\_ROI,IEV3}$	50	0.001	0.246	-0.373	Shape
$SV_{V,IEV3}$	51	-0.03	0.336	-0.36	Poros
$SF_{Tri,minB,V\_ROI,IEV2}$	52	-0.002	0.049	-0.359	Shape
$SF_{PolyArea,V,IEV3}$	53	0	0.058	-0.347	Shape
$SF_{Sph,minB,F,V,IEV3}$	54	0.001	0.05	-0.346	Shape
$SF_{SemiSph,minB,F,V,IEV2}$	55	0.001	0.079	-0.346	Shape
$SF_{Rect,minB,F,V,IEV3}$	56	0.001	0.05	-0.346	Shape
$d_{eqSph,V,IEV2}$	57	-0.018	0.346	-0.183	Size
$\Psi_{lR,max20,V\_ROI,IEV3}$	58	0.001	0.168	-0.345	Shape
$SF_{Tri,minB,F,V,IEV3}$	59	0.001	0.044	-0.344	Shape
$SF_{Tri,minB,V\_ROI,IEV1}$	60	-0.002	0.341	-0.041	Shape
$SF_{SemiSph,minB,F,V,IEV3}$	61	0.002	0.057	-0.341	Shape
$EX_{V,IEV1}$	62	-0.008	0.336	-0.333	Shape
AR_{V}	63	0	0.336	-0.095	Shape
$SV_{V,IEV1}$	64	-0.028	0.323	-0.335	Poros
$SF_{Rect,minB,V,IEV2}$	65	-0.002	0.334	-0.058	Shape
$SF_{SemiSph,minB,F,V\_ROI,IEV2}$	66	0.001	0.332	-0.04	Shape
$SF_{Tri,minB,F,V,IEV2}$	67	0	0.071	-0.33	Shape
$V_{V,IEV3}$	68	-0.026	0.326	-0.307	Size
$SF_{Rect,minB,F,V,IEV2}$	69	0.001	0.101	-0.323	Shape
$SF_{Sph,minB,F,V\_ROI,IEV2}$	70	0	0.039	-0.32	Shape
$SF_{SemiSph,minB,V,IEV1}$	71	-0.001	0.067	-0.318	Shape
$V_{V,IEV1}$	72	-0.021	0.315	-0.316	Size
$V_{CH,V,IEV2}$	73	-0.002	0.312	-0.043	Shape
$ECC_{V,IEV1}$	74	-0.002	0.212	-0.311	Shape

$Z_{A,V\_ROI,IEV3}$	75	-0.002	0.309	-0.106	Size
$SF_{PolyArea,V\_ROI,IEV2}$	76	-0.002	0.04	-0.309	Shape
$V_{V\_ROI,IEV2}$	77	-0.002	0.04	-0.306	Size
$SF_{Elps,IM,dMin,V\_ROI,IEV2}$	78	-0.001	0.036	-0.3	Size
EX_{V}	79	-0.03	0.298	-0.261	Shape
SV	80	-0.028	0.292	-0.259	Poros
$V_{maxFeretSph,F,V}$	81	-0.02	0.291	-0.26	Shape
V_{V}	82	-0.031	0.286	-0.263	Size
$V_{FA,V,IEV3}$	83	0	0.054	-0.281	Poros
$SF_{Elps,SA,r3,V}$	84	-0.001	0.078	-0.275	Size
$SF_{Sph,minB,F,V\_ROI,IEV1}$	85	0	0.274	-0.05	Shape
$SF_{PolyArea,V\_ROI,IEV1}$	86	-0.002	0.269	-0.036	Shape
$V_{V\_ROI,IEV1}$	87	-0.002	0.266	-0.035	Size
$SF_{Rect,minB,V\_ROI,IEV2}$	88	-0.002	0.037	-0.264	Shape
$V_{CH,V\_ROI,IEV2}$	89	-0.002	0.035	-0.248	Shape
$SF_{Tri,minB,V,IEV1}$	90	-0.001	0.081	-0.238	Shape
$V_{CH,V\_ROI,IEV1}$	91	-0.002	0.235	-0.032	Shape
$EX_{V\_ROI,IEV1}$	92	0	0.228	-0.048	Shape
$\beta_{E,V}$	93	0.002	0.191	-0.228	Orientation
$SF_{Rect,minB,V\_ROI,IEV1}$	94	-0.002	0.225	-0.029	Shape
$SF_{Elps,IM,dMin,V\_ROI,IEV1}$	95	-0.001	0.221	-0.028	Size
$SF_{SemiSph,minB,F,V,IEV1}$	96	0	0.205	-0.129	Shape
$l_{BB,2,V\_ROI,IEV3}$	97	-0.002	0.059	-0.202	Size
$EX_{V\_ROI,IEV2}$	98	0	0.025	-0.202	Shape
$l_{BB,1,V\_ROI,IEV3}$	99	-0.001	0.199	-0.055	Size
$d_{eqSph,V,IEV3}$	100	-0.009	0.16	-0.198	Size
$ECC_{V,IEV3}$	101	0	0.181	-0.196	Shape

$l_{BB,1,V\_ROI,IEV2}$	102	-0.001	0.188	-0.054	Size
$d_{eqSph,V,IEV1}$	103	-0.013	0.154	-0.187	Size
$SF_{Sph,minB,F,V,IEV1}$	104	0.001	0.121	-0.187	Shape
$l_{BB,2,V,IEV3}$	105	-0.002	0.184	-0.064	Size
$SF_{Sph,minB,r,V\_ROI}$	106	0.007	0.081	-0.183	Size
$Z_{A,V\_ROI,IEV1}$	107	-0.002	0.097	-0.181	Shape
$SF_{Elps,IM,dMin,V,IEV1}$	108	0.003	0.07	-0.178	Size
$SF_{PolyArea,V,IEV1}$	109	-0.001	0.067	-0.172	Shape
$l_{BB,1,V\_ROI,IEV1}$	110	-0.001	0.165	-0.036	Size
$d_{eqSph,V\_ROI,IEV2}$	111	-0.001	0.021	-0.165	Size
$SF_{SemiSph,minB,F,V\_ROI,IEV1}$	112	0	0.026	-0.165	Shape
$SF_{Elps,SA,chi2,V\_ROI}$	113	-0.004	0.165	-0.085	Shape
$SF_{Rect,minB,V,IEV1}$	114	0	0.091	-0.161	Shape
$ECC_{V\_ROI,IEV3}$	115	-0.001	0.154	-0.036	Shape
$l_{BB,1,V,IEV2}$	116	0.001	0.057	-0.151	Size
$l_{BB,2,V,IEV1}$	117	0.001	0.149	-0.111	Size
$SF_{SemiSph,minB,V,IEV3}$	118	-0.002	0.146	-0.147	Shape
$ECC_{V\_ROI,IEV1}$	119	0	0.037	-0.142	Shape
γ_{E,V_ROI}	120	0	0.029	-0.138	Orientation
$SF_{Sph,minB,V,IEV1}$	121	-0.001	0.1	-0.136	Shape
$SF_{Elps,IM,dMin,V,IEV3}$	122	0.001	0.054	-0.128	Size
$SF_{Elps,IM,dMax,V,IEV1}$	123	0.002	0.051	-0.127	Size
$d_{eqSph,V\_ROI,IEV1}$	124	-0.001	0.126	-0.017	Size
$SF_{Rect,minB,F,V,IEV1}$	125	0.001	0.1	-0.126	Shape
Ψ_{gl,V_ROI}	126	0.002	0.056	-0.118	Shape
$SF_{SemiSph,minB,F,V\_ROI,IEV3}$	127	-0.001	0.11	-0.025	Shape
$l_{BB,1,V,IEV3}$	128	-0.001	0.055	-0.106	Size

$l_{BB,2,V,IEV2}$	129	0	0.048	-0.103	Size
$V_{CH,V,IEV3}$	130	-0.002	0.06	-0.102	Shape
$EX_{V\_ROI,IEV3}$	131	0	0.034	-0.101	Shape
$A_{Sf,V\_ROI,IEV3}$	132	-0.002	0.101	-0.033	Surface

S1.5. ReliefF Feature Selection Results

Table S6: ReliefF feature ranking of 74 extracted structural features linked to pellet size, shape, surface and orientation attributes. The importance of individual features is expressed by the calculated ReliefF weights which range from -1 to 1 for low and high importance, respectively. Further, the list includes p-values from a two-sample t-test (H_0 of equal means) and F-scores to indicate class separation for individual features but failing to address important feature-feature dependencies

Feature	Rank	Weight	p-Value	F-Score	Category
**$V_{maxFeretSph,F,V\_ROI}$**	**1**	**0.536**	**0**	**21.524**	**Shape**
$V_{maxFeretSph,F,V\_ROI,CH}$	2	0.509	0	18.418	Shape
EX_{V_ROI}	3	0.454	0	122.604	Shape
$SF_{Sph,minB,F,V\_ROI,IEV3}$	4	0.45	0	36.905	Shape
AR_{V_ROI}	5	0.382	0	74.827	Shape
**$SF_{Elps,SA,r3,V\_ROI}$**	**6**	**0.361**	**0**	**69.653**	**Size**
**$SV_{CH,V\_ROI,IEV1}$**	**7**	**0.306**	**0**	**77.498**	**Surface**
$SF_{Tri,minB,F,V\_ROI,IEV1}$	8	0.305	0	22.544	Shape
$SF_{Rect,minB,F,V\_ROI,IEV1}$	9	0.299	0	75.071	Shape
$SV_{CH,V\_ROI,IEV3}$	10	0.296	0	69.095	Surface

$SV_{CH,V\_ROI,IEV2}$	11	0.283	0	61.428	Surface
$SF_{Tri,minB,F,V\_ROI,IEV3}$	12	0.254	0	22.412	Shape
$SF_{Elps,IM,dMin,V\_ROI,IEV3}$	13	0.25	0	20.187	Size
$SF_{Tri,minB,F,V\_ROI,IEV2}$	14	0.247	0	14.864	Shape
$SF_{Rect,minB,F,V\_ROI,IEV3}$	15	0.246	0	43.947	Shape
$SF_{SemiSph,minB,V\_ROI,IEV3}$	16	0.223	0	28.755	Shape
$SF_{Tri,minB,F,V,IEV1}$	17	0.218	0.003	9.712	Shape
$SF_{Rect,minB,F,V\_ROI,IEV2}$	18	0.205	0.001	16.479	Shape
d_{eqSph,V_ROI}	19	0.19	0	28.666	Size
$d_{eqSph,V}$	20	0.182	0	21.317	Size
$d_{eqSph,V\_ROI,IEV3}$	21	0.175	0	12.443	Size
$SF_{Tri,minB,V,IEV3}$	22	0.173	0	19.858	Shape
$SF_{Tri,minB,V\_ROI,IEV3}$	23	0.172	0	19.62	Shape
$l_{BB,min,V}$	24	0.167	0	30.148	Size
l_{BB,min,V_ROI}	25	0.167	0	30.148	Size
$SF_{PolyArea,V\_ROI,IEV3}$	26	0.164	0	18.712	Shape
V_{V_ROI}	27	0.149	0	14.165	Size
$V_{V\_ROI,IEV3}$	28	0.148	0	7.965	Size
$SF_{Rect,minB,V,IEV3}$	29	0.146	0	17.231	Shape
$SF_{Rect,minB,V\_ROI,IEV3}$	30	0.145	0	17.019	Shape
$V_{CH,V\_ROI,IEV3}$	31	0.137	0	7.57	Shape
V_{CH,V_ROI}	32	0.132	0	13.305	Size
$SF_{Elps,SA,r2,V}$	33	0.129	0	22.872	Size
SV_{CH,V_ROI}	34	0.126	0.7	0.132	Surface
$SF_{Elps,SA,r1,V}$	35	0.122	0	3.298	Size
$SF_{Elps,SA,r2,V\_ROI}$	36	0.121	0	21.183	Size
$A_{Sf,V\_ROI,IEV1}$	37	0.112	0	8.916	Surface

A_{Sf,V_ROI}	38	0.111	0	14.598	Surface
$SF_{Elps,SA,r1,V\_ROI}$	39	0.109	0	3.712	Size
$l_{IB,y}$	40	0.106	0	15.145	Size
$A_{Sf,V\_ROI,IEV2}$	41	0.104	0	6.45	Surface
$l_{BB,2,V\_ROI,IEV1}$	42	0.099	0.044	0.428	Size
$l_{IB,z}$	43	0.097	0	11.666	Size
$l_{BB,2,V\_ROI,IEV2}$	44	0.093	0.027	0.464	Size
$l_{IB,x}$	45	0.092	0	13.497	Size
$l_{BB,1,V,IEV1}$	46	0.089	0.034	0.314	Size
$d_{maxFeret,V}$	47	0.083	0	5.021	Size
$V_{IB,V}$	48	0.078	0	10.256	Size
$SF_{Elps,IM,dMax,V\_ROI,IEV1}$	49	0.072	0	5.468	Size
l_{BB,max,V_ROI}	50	0.07	0	8.199	Size
$l_{BB,max,V}$	51	0.07	0	8.199	Size
$SF_{Elps,IM,dMax,V,IEV3}$	52	0.069	0	4.028	Size
$SF_{Elps,IM,dMax,V\_ROI,IEV3}$	53	0.064	0	4.169	Size
$x_{c,V,IEV1}$	54	0.06	0	2.651	Shape
$y_{c,V,IEV2}$	55	0.059	0	2.692	Shape
$x_{c,V\_ROI,IEV3}$	56	0.059	0	2.771	Shape
$x_{c,V\_ROI,IEV2}$	57	0.058	0	2.608	Shape
$y_{c,V\_ROI,IEV3}$	58	0.057	0	2.639	Shape
$x_{c,V,IEV2}$	59	0.057	0	2.644	Shape
$x_{c,V\_ROI,IEV1}$	60	0.057	0	2.698	Shape
$y_{c,V\_ROI,IEV2}$	61	0.056	0	2.75	Shape
$y_{c,V,IEV1}$	62	0.056	0	2.709	Shape
$x_{c,V,IEV3}$	63	0.056	0	2.79	Shape
$SF_{Sph,minB,V\_ROI,IEV3}$	64	0.056	0	5.271	Shape

$SF_{Sph,minB,V,IEV3}$	65	0.056	0	5.315	Shape
$SF_{Elps,IM,dMax,V,IEV2}$	66	0.054	0	4.164	Size
$y_{c,V,IEV3}$	67	0.054	0	2.691	Shape
$y_{c,V\_ROI,IEV1}$	68	0.053	0	2.779	Shape
$SF_{Sph,minB,V\_ROI,IEV1}$	69	0.051	0	7.13	Shape
$SF_{Elps,IM,dMax,V\_ROI,IEV2}$	70	0.047	0	4.515	Size
$SF_{Sph,minB,V\_ROI,IEV2}$	71	0.043	0	5.474	Shape
$SF_{Sph,minB,V,IEV2}$	72	0.041	0	5.654	Shape
α_{E,V_ROI}	73	0.037	0.861	0.001	Orientation
β_{E,V_ROI}	74	0.033	0.732	0.007	Orientation

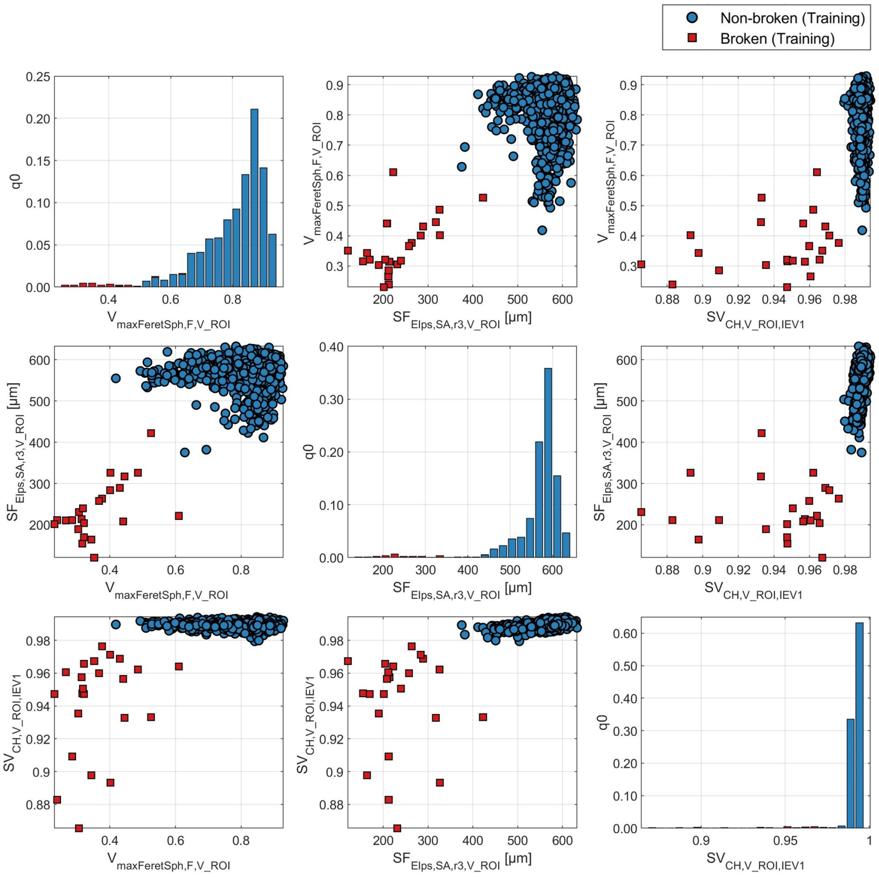

Figure S3: Plotmatrix of the training dataset (DTR) with feature combinations of $SF_{maxFeretSph,F,V\_ROI}$, $SF_{Elps,SA,r3,V\_ROI}$ and $SV_{CH,V\_ROI,IEV1}$.

References

[1] F. Doerr, I. Oswald, A. Florence, Quantitative investigation of particle formation of a model pharmaceutical formulation using single droplet evaporation experiments and X-ray tomography, Advanced Powder Technology 29 (12) (2018) 2996–3006. doi:10.1016/j.apt.2018.09.027.

[2] Y. Petrov, Ellipsoid fit (MATLAB FileExchange) (2015).
URL https://uk.mathworks.com/matlabcentral/fileexchange/24693-ellipsoid-fit

[3] J. D'Errico, A suite of minimal bounding objects (MATLAB FileExchange) (2014).
URL https://uk.mathworks.com/matlabcentral/fileexchange/34767-a-suite-of-minimal-bounding-objects

[4] H. Wadell, Volume, Shape, and Roundness of Quartz Particles, The Journal of Geology 43 (3) (1935) 250–280. doi:10.1086/624298.

[5] J. Zheng, R. Hryciw, Traditional soil particle sphericity, roundness and surface roughness by computational geometry, Géotechnique 65 (6) (2015) 494–506. doi:10.1680/geot.14.P.192.

[6] A. Tahmasbi, F. Saki, S. B. Shokouhi, Classification of benign and malignant masses based on Zernike moments, Computers in Biology and Medicine 41 (8) (2011) 726–735. doi:10.1016/j.compbiomed.2011.06.009.
